# Supplementary material for: ARGONAUTE10 controls cell fate specification and formative cell divisions in the Arabidopsis root
Source: EMBO J. 2024 Apr 2;43(9):7. doi: 10.1038/s44318-024-00072-x (PMC11066080; doi:10.1038/s44318-024-00072-x)
Supplement: Supplementary file 1 — Appendix [file 44318_2024_72_MOESM1_ESM.pdf]

Appendix for **ARGONAUTE10 controls cell fate specification and formative cell divisions in the Arabidopsis root**

Nabila El Arbi, Ann-Kathrin Schürholz, Marlene U. Handl, Liese Schnurbusch, Alexei Schiffner, Inés Hidalgo Prados, Christian Wenzl, Xin'Ai Zhao, Jian Zeng, Jan U. Lohmann, Sebastian Wolf

Table of Content

Appendix Figures

Appendix Figure S1. *sgo1* is a recessive mutation. p. 2

Appendix Figure S2. *sgo1* shows enhanced resistance to water-limited conditions. p. 3

Appendix Figure S3. Starch accumulation as indicated by Lugol's staining is not affected in AGO10 mutants. p. 5

Appendix Tables

Appendix Table S1. Plant material used in this study. p. 6

Appendix Table S2. Oligonucleotides used in this study. p. 7

Appendix Table S3. GreenGate constructs p. 10

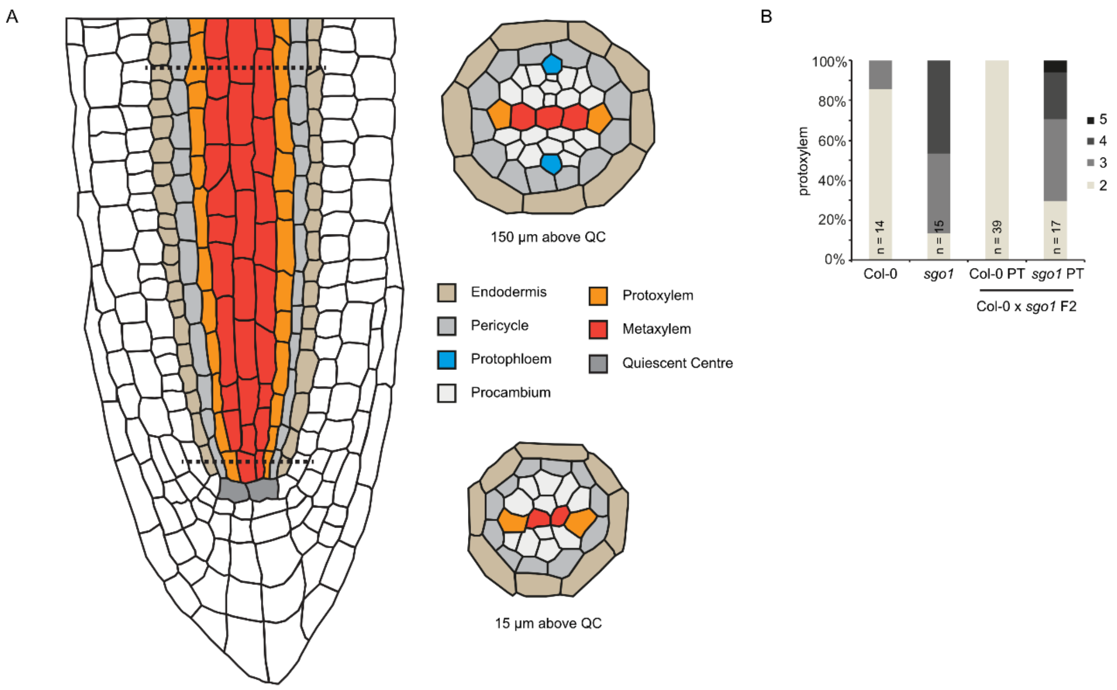

Appendix Figure S1. Related to Figure 1. *sgo1* is a recessive mutation. (A) Schematic representation of the Arabidopsis root meristem (left) and cross sections through the vascular cylinder and the endodermis 15 µm and 150 µm distance from the quiescent centre (QC, right). (B) Protoxylem quantification of Col-0, *sgo1* and segregation of protoxylem phenotype in F2 population derived from a cross between the two genotypes. Graph depicts frequency of roots with the indicated number of protoxylem cells. PT = phenotype.

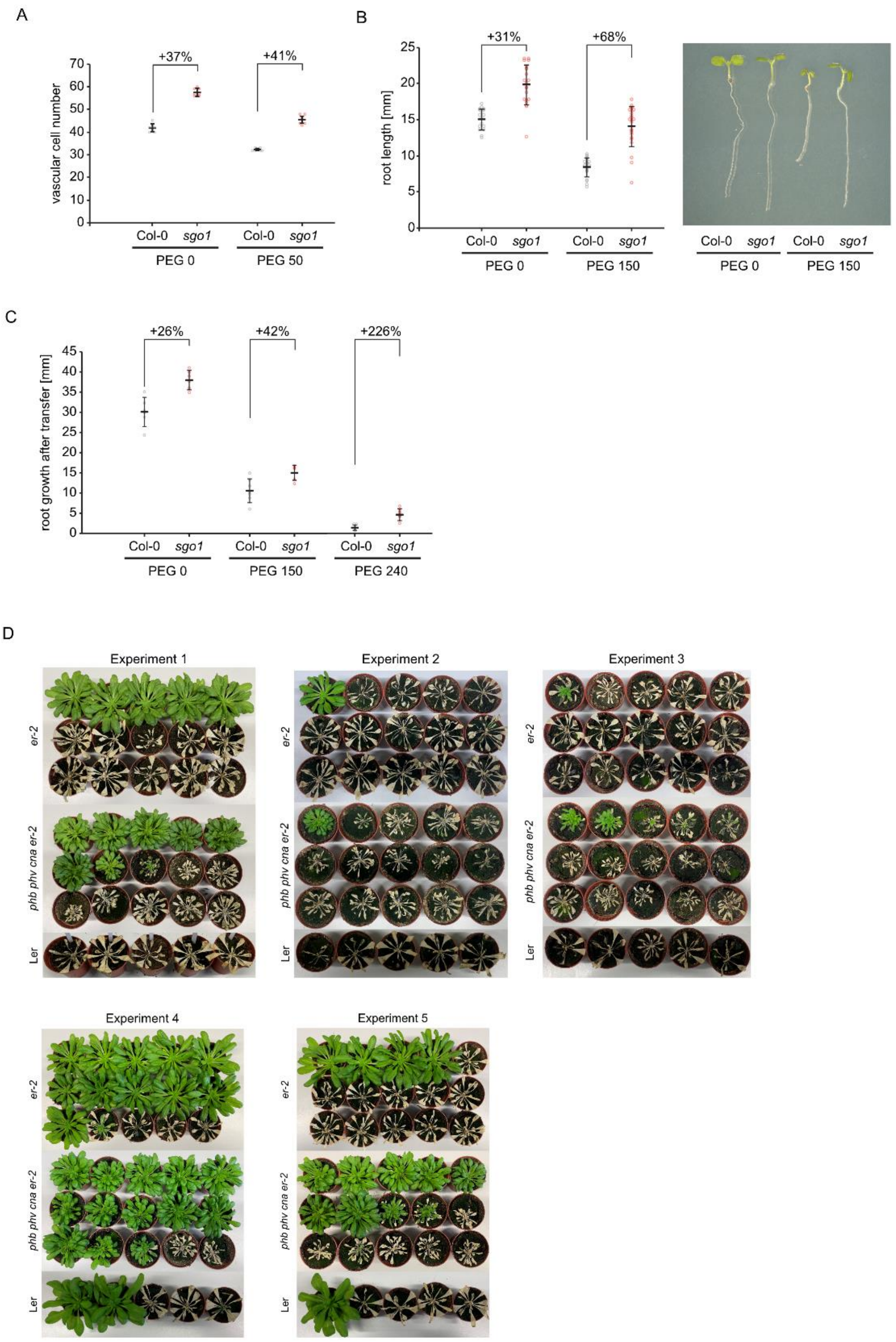

Appendix Figure S2. Related to Figure 6. *sgo1* shows enhanced resistance to water-limited conditions. (A) Meristematic vascular cell number 150 µm from the QC of Col-0 and sgo1 plants seven days after germination on control medium or medium simulating water

deficit by addition of 150 g/L PEG8000. Graph indicates mean ± s.d., individual data points are indicated. (B) Root length of plants grown on control medium of medium simulating water deficit by addition of 150 g/L PEG8000 at seven days after germination. Image depicts representative seedlings. Graph indicates mean ± s.d., individual data points are indicated. (C) Root growth 11 days after transfer of five-day old seedlings to medium infused with the indicated amount of PEG8000. Graph indicates mean ± s.d., individual data points are indicated. (D) *phb phv can* mutants show slightly increased survival in drought conditions. Watering of four-week-old plants was stopped for two weeks and then recommenced. Images were taken 10 days after initial re-watering. Plants were grown randomly dispersed in the same tray.

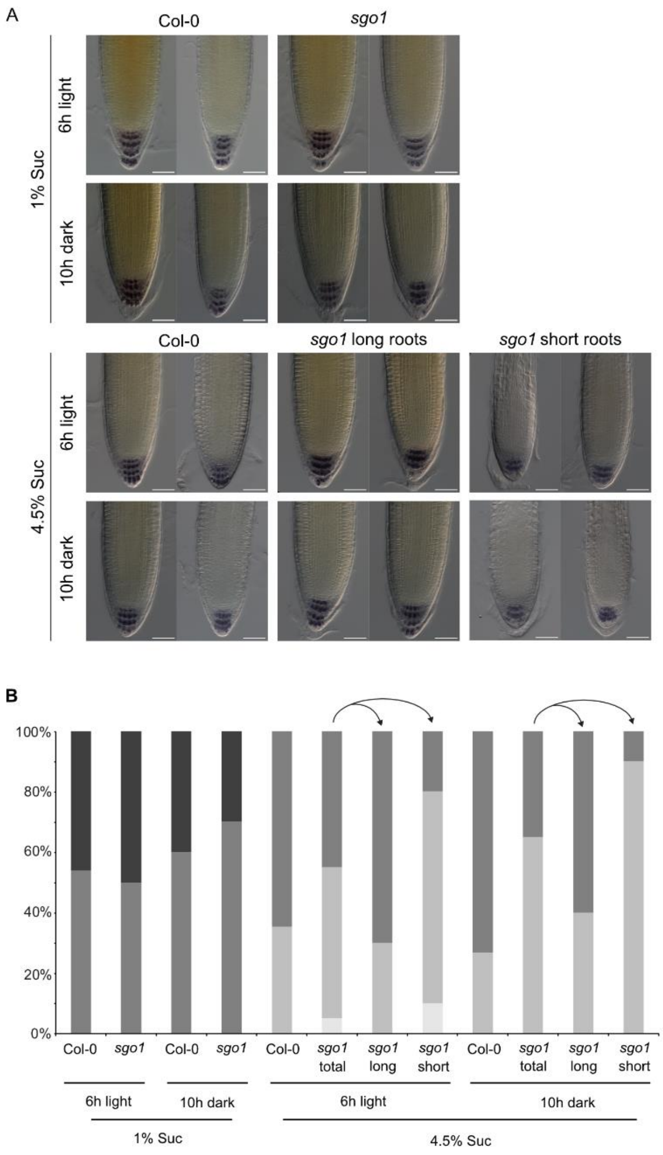

Appendix Figure S3. Related to Figure 6. Starch accumulation as indicated by Lugol's staining is not affected in AGO10 mutants. (A) Accumulation of starch granules in root tips of Col-0 and *sgo1* plants was visualized with Lugol staining after 6 hours of light exposure and directly before light exposure started in the morning of the sixth day after germination. (B) Quantification of cell layers with starch granules in roots grown in the indicated conditions.

Arabidopsis: Col-0 WT	
Arabidopsis: L*er*	
Arabidopsis: *sgo1*	This study
Arabidopsis: *ago10-1*	Takeda et al., 2008
Arabidopsis: *phb-13*	Prigge et al., 2005
Arabidopsis: *phv-11*	Prigge et al., 2005
Arabidopsis: *cna-2*	Prigge et al., 2005
Arabidopsis: *phb-11 phv-13 cna-2*	Prigge et al., 2005
Arabidopsis: *pZLL-GFP*	Palovaara et al., 2017
Arabidopsis: *pAHP6:erGFP*	Mähönen et al., 2006
Arabidopsis: *pAHP6:erGFP* (*sgo1*)	This study
Arabidopsis: *pTMO5:NLS-3xGFP*	Schlereth et al., 2010
Arabidopsis: *pTMO5:NLS-3xGFP* (*sgo1*)	This study
Arabidopsis: *pDR5v2:erYFP*	Ma et al., 2019
Arabidopsis: *pDR5v2:erYFP* (*sgo1*)	This study
Arabidopsis: *pMIR165a:GFP*	Carlsbecker et al., 2010
Arabidopsis: *pTCSn:GFP-NLSm pUB10:RFP-NLS*	This study
Arabidopsis: *pTCSn:GFP-NLSm pUB10:RFP-NLS* (*sgo1*)	This study
Arabidopsis: *zll-3*	Moussian 1998

Appendix Table S1. Plant material used in this study.

Oligo name	Sequence	Purpose
CER448567_F	ATA GAA AGG TTT GAG GGG GC	Bulked segregant analysis
CER448567_R	TGC GAA GAA CCA CTA AAC CC	Bulked segregant analysis
F9L1_F	CTC GGA AAT TCT TAG CTT TC	Bulked segregant analysis
F9L1_R	TTA TAA CTT GCC CAA AGC GAA	Bulked segregant analysis
F1K23ind38_F	GGA TTG AAC ATA GGG AAG GGG	Bulked segregant analysis
F1K23ind38_R	GAT CTG TAT CTG AAA CCT GGG	Bulked segregant analysis
CER464787-Indel-44_F	TTT GAA CTA ACC TTC TGA GG	Bulked segregant analysis
CER464787-Indel-44_R	CAT GTT GAT GAT TCA ATT GC	Bulked segregant analysis
F6D8ind94_F	CCG TTA CCC CCA TAC GAA CG	Bulked segregant analysis
F6D8ind94_R	TCG TGA GGT TAT GCC GAT CC	Bulked segregant analysis
F5I14_F	CTG CCT GAA ATT GTC GAA AC	Bulked segregant analysis
F5I14_R	GGC ATC ACA GTT CTG ATT CC	Bulked segregant analysis
CER459153_F	TCG TGA CCA AAT CCT GAA CA	Bulked segregant analysis
CER459153_R	TGT CCA AGT AAT GCC GTG AG	Bulked segregant analysis
CER466780_F	GAA CCC TTA TAA TAT GGC TGG C	Bulked segregant analysis
CER466780_R	GGA AGT ATT CCC AAG ACA AGG	Bulked segregant analysis
MSAT2-36_F	GAT CTG CCT CTT GAT CAG C	Bulked segregant analysis
MSAT2-36_R	CCA AGA ACT CAA AAC CGT T	Bulked segregant analysis
F3N11_F	GTT AAA GCG AGG ACG ATT GG	Bulked segregant analysis
F3N11_R	AGA TAC TGT CGC CAT CAA GG	Bulked segregant analysis
T2P4_F	ACT AGT CCC ACT GTC GAT C	Bulked segregant analysis
T2P4_R	GTT ACT TCG TAA GTC CCT AC	Bulked segregant analysis
MSAT2-9_F	TAA AAG AGT CCC TCG TAA AG	Bulked segregant analysis
MSAT2-9_R	GTT GTT GTT GTG GCA TT	Bulked segregant analysis
nga172_F	AGC TGC TTC CTT ATA GCG TCC	Bulked segregant analysis
nga172_R	CAT CCG AAT GCC ATT GTT C	Bulked segregant analysis
CER455386_F	CTC TTT TGG CTC GGA CAA G	Bulked segregant analysis
CER455386_R	GTT GTA ATC GGG AAA ATG C	Bulked segregant analysis
CER455914_F	GGA GCA GAG AAA GAG AC	Bulked segregant analysis
CER455914_R	GAG GAA GGA CAA CAT GGC	Bulked segregant analysis
CER456071-Indel-35_F	AGC CAT AGG TAA TGT CCA CG	Bulked segregant analysis
CER456071-Indel-35_R	CTC GCG GAT GAG TAT CAT CC	Bulked segregant analysis

CER470441_F	GCT AAC AGG GAT ATC AAA TGT GC	Bulked segregant analysis
CER470441_R	CGG ACG AGC TGA CAC TTG TA	Bulked segregant analysis
CER470172_F	GTA AAA CTC CTC CTC TGG GG	Bulked segregant analysis
CER470172_R	TGT AAT CGT GGC GGA ACG GG	Bulked segregant analysis
CER459609_F	TCG CTT TTG AAG ATT TGT GC	Bulked segregant analysis
CER459609_R	GGG AGC TTC TCA GTG GTC TG	Bulked segregant analysis
nga8_F	GAG GGC AAA TCT TTA TTT CGG	Bulked segregant analysis
nga8_R	TGG CTT TCG TTT ATA AAC ATC C	Bulked segregant analysis
FCA0ind25_F	AAG CCA ACT ATT GCC AAG GG	Bulked segregant analysis
FCA0ind25_R	TCA CTG CCC TTT ACT CCG GT	Bulked segregant analysis
F7J7-47_F	TGG TGA AGA GCT TAG TTG ATG A	Bulked segregant analysis
F7J7-47_R	TCA CTA GAT ATC TCT AGT GGC T	Bulked segregant analysis
CER451534_F	AGC TAC GGT GGA GTG TAA TTT CGT	Bulked segregant analysis
CER451534_R	GCT GAT ACT TGC TTT CGC TTT GCA G	Bulked segregant analysis
CER459444_F	AGT AGC ATC GTA GCT CCT AGG	Bulked segregant analysis
CER459444_R	GTT GTA TAC GTG CAC GTT CCC	Bulked segregant analysis
CER456519_F	TGC TAA AAT ATA AAA CTT CC	Bulked segregant analysis
CER456519_R	TTA TGC AGA TGT ATG AGG CC	Bulked segregant analysis
nga151_F	GTT TTG GGA AGT TTT GCT GG	Bulked segregant analysis
nga151_R	CAG TCT AAA AGC GAG AGT ATG ATG	Bulked segregant analysis
nga139_F	GGT TTC GTT TCA CTA TCC AGG	Bulked segregant analysis
nga139_R	AGA GCT ACC AGA TCC GAT GG	Bulked segregant analysis
T26D22-IND52/CER459812_F	TCC CAC GAA GAG AGA AGT GC	Bulked segregant analysis
T26D22-IND52/CER459812_R	CTA TTT GCT TAT GAA GGT GTC C	Bulked segregant analysis
CER456772_F	CCA TGT GAC ATG CAC TTA CAC	Bulked segregant analysis
CER456772_R	ACC ATT CTC TAC CAC TCC AC	Bulked segregant analysis
K6M13ind33/CER454758_F	ATA GAT GAG ATC CAC TTG CC	Bulked segregant analysis
K6M13ind33/CER454758_R	ACA AAC TGT TGC TGT GGG AG	Bulked segregant analysis
MBK5ind35/CER455203_F	ATT CTC GGA CCA GGC TTC AT	Bulked segregant analysis
MBK5ind35/CER455203_R	AAA GAA CAG CTA CTG CGT GC	Bulked segregant analysis
nga151_R	CAG TCT AAA AGC GAG AGT ATG ATG	Bulked segregant analysis
nga139_F	GGT TTC GTT TCA CTA TCC AGG	Bulked segregant analysis
nga139_R	AGA GCT ACC AGA TCC GAT GG	Bulked segregant analysis

T26D22-IND52/CER459812_F	TCC CAC GAA GAG AGA AGT GC	Bulked segregant analysis
T26D22-IND52/CER459812_R	CTA TTT GCT TAT GAA GGT GTC C	Bulked segregant analysis
At5g43810_sgo1_dCAPS_F	GTTTCCAGTAGATGAGAACTG	dCAPS marker (HindIII)
At5g43810_sgo1_dCAPS_R	AACAAACAAACCAGCAGTTTACGGTTAAGCT	dCAPS marker (HindIII)
At5g47720_Col_dCAPS_F	CTGGGATTAGATCCTgtaagtc	dCAPS marker (HinfI)
At5g47720_Col_dCAPS_R	CCCAATGGATGTCCCAGTGAAACAGCGACT	dCAPS marker (HinfI)
At5g51560_CAPS_F	ctctcgaggcaaatgtgtaat	CAPS marker (XmnIII)
At5g51560_CAPS_R	AGGGTTGAAGAAATGACATCC	CAPS marker (XmnI)
qPHB-F	CTTTGGTAGTGGCGTGCTTT	qRT-PCR
qPHB-R	GCCCATTCAGATCGGTGTTC	qRT-PCR
qPHV-F	CCAAGATCATGCAGCAGGGA	qRT-PCR
qPHV-R	CGCTTGCTCATACGAAACCG	qRT-PCR
qCNA-F	AGAATGTTCCTCCGGCGATC	qRT-PCR
qCNA-R	TGCCCTCCAAATCCTCCAAC	qRT-PCR
qPri-MIR166b-F	TGTCTGGCTCGAGGACTCTT	qRT-PCR
qPri-MIR166b-R	TCCGACGACACTAAAACCCT	qRT-PCR
qmiR166-F	TCGCTTCGGACCAGGCTTCA	qRT-PCR
qmiR166-R	GTGCAGGGTCCGAGGT	qRT-PCR
miR166stem-loop	GTCGTATCCAGTGCAGGGTCCGAGG TATTCGCACTGGATACGACGGGGAA	Stem-loop RT primer
ACT2-F	CTAAGCTCTCAAGATCAAAGGCTTA	qRT-PCR
ACT2-R	ACTAAAACGCAAAACGAAAGCGGTT	qRT-PCR
A04555	AACAGGTCTCCGGCTAAAAGCTTGACTAGT CAAAGATC	Cloning of pGGA043 (pTCSn)
A04630	AAC AGG TCT CaT GTT TCT CCA AAT GAA ATG AAC TTC	Cloning of pGGA043 (pTCSn)
A03821	AAC AGG TCT CAA ACA ATG GTG AGC AAG GGC GAG	Cloning of pGGB007
A02228	AAT TCG GTC TCA AGC CAG CAA TTG CTG CGG CAG C	Cloning of pGGB007
A02530	AAC AGG TCT CAG GCT CCA TGG TGA GCA AGG GCG AGG A	Cloning of pGGC023
A02531	AAC AGG TCT CAC TGA CTT GTA CAG CTC GTC CAT G	Cloning of pGGC023

Appendix Table S2. Oligonucleotides used in this study.

**pCW178**	**pTCSn:2xVenus-NLS**		
pGGA043	pTCSn	A04555	A04630
pGGB007	Venus-linker	A03821	A02228
pGGC023	Venus	A02530	A02531
pGGD007	Linker-NLS	Lampropoulos et al., 2013	
pGGE001	*RBCS* terminator	Lampropoulos et al., 2013	
pGGF001	pMAS:BastaR:tMAS	Lampropoulos et al., 2013	
pGGZ0003	destination vector	Lampropoulos et al., 2013	

**pCW066**	**pUBQ10:3xmCherry-NLS**		
pGGA006	UBQ10 (At4g05320)promoter	Lampropoulos et al., 2013	
pGGB003	B-Dummy	Lampropoulos et al., 2013	
pGGC026	3xmCherry	Lampropoulos et al., 2013	
pGGD007	Linker-NLS	Lampropoulos et al., 2013	
pGGE009	UBQ10 terminator	Lampropoulos et al., 2013	
pGGF007	pNOS:KanR:tNOS	Lampropoulos et al., 2013	
pGGZ003	destination vector	Lampropoulos et al., 2013	

Appendix Table S3. **Overview of constructs generated with GreenGate cloning (Lampropoulos et al., 2013)**
