# Supplementary material for: ARGONAUTE10 controls cell fate specification and formative cell divisions in the Arabidopsis root
Source: EMBO J. 2024 Apr 2;43(9):7. doi: 10.1038/s44318-024-00072-x (PMC11066080; doi:10.1038/s44318-024-00072-x)
Supplement: Supplementary file 3 — Movie EV1 [file 44318_2024_72_MOESM3_ESM.zip › Movie EV1/Movie EV1.docx]

Movie EV1. 3D reconstruction of a Col-0 plant imaged after an EdU-free period of 6 hours following a 40-minute EdU pulse.
