# Supplementary material for: ARGONAUTE10 controls cell fate specification and formative cell divisions in the Arabidopsis root
Source: EMBO J. 2024 Apr 2;43(9):7. doi: 10.1038/s44318-024-00072-x (PMC11066080; doi:10.1038/s44318-024-00072-x)
Supplement: Supplementary file 4 — Movie EV2 [file 44318_2024_72_MOESM4_ESM.zip › Movie EV2/Movie EV2.docx]

Movie EV2. 3D reconstruction of a *sgo1* plant imaged after an EdU-free period of 6 hours following a 40-minute EdU pulse.
