# Supplementary material for: ARGONAUTE10 controls cell fate specification and formative cell divisions in the Arabidopsis root
Source: EMBO J. 2024 Apr 2;43(9):7. doi: 10.1038/s44318-024-00072-x (PMC11066080; doi:10.1038/s44318-024-00072-x)
Supplement: Supplementary file 5 — Movie EV3 [file 44318_2024_72_MOESM5_ESM.zip › Movie EV3/Movie EV3.docx]

Movie EV3. 3D reconstruction of a*TMO5:NLS-3xGFP* root showing the typical reporter expression mostly confined to five xylem precursor cells.
