# Supplementary material for: ARGONAUTE10 controls cell fate specification and formative cell divisions in the Arabidopsis root
Source: EMBO J. 2024 Apr 2;43(9):7. doi: 10.1038/s44318-024-00072-x (PMC11066080; doi:10.1038/s44318-024-00072-x)
Supplement: Supplementary file 6 — Movie EV4 [file 44318_2024_72_MOESM6_ESM.zip › Movie EV4/Movie EV4.docx]

Movie EV4. 3D reconstruction of the *TMO5:NLS-3xGFP* signal in an *sgo1* root, displaying expanded reporter expression compared to Col-0.
