# Supplementary material for: ARGONAUTE10 controls cell fate specification and formative cell divisions in the Arabidopsis root
Source: EMBO J. 2024 Apr 2;43(9):7. doi: 10.1038/s44318-024-00072-x (PMC11066080; doi:10.1038/s44318-024-00072-x)
Supplement: Supplementary file 7 — Movie EV5 [file 44318_2024_72_MOESM7_ESM.zip › Movie EV5/Movie EV5.docx]

Movie EV5. 3D reconstruction of the *pTCSn:2xVenus-NLS* reporter signal in the Col-0 background.
