# Supplementary figures and images for: ARGONAUTE10 controls cell fate specification and formative cell divisions in the Arabidopsis root
Source: EMBO J. 2024 Apr 2;43(9):7. doi: 10.1038/s44318-024-00072-x (PMC11066080; doi:10.1038/s44318-024-00072-x)

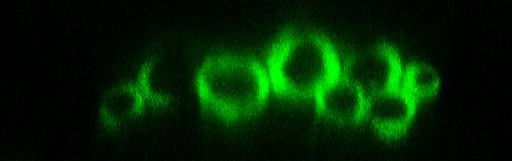

Supplement: Supplementary file 9 — Source data Fig. 1 [file 44318_2024_72_MOESM9_ESM.zip › Figure 1/1A/sgo1_projection resliced.tif]

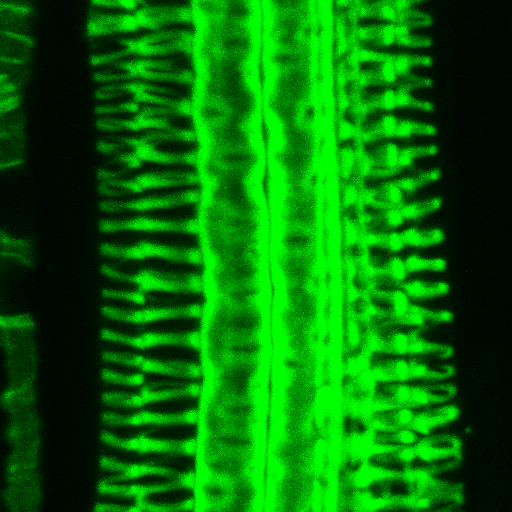

Supplement: Supplementary file 9 — Source data Fig. 1 [file 44318_2024_72_MOESM9_ESM.zip › Figure 1/1A/sgo1_projection.tif]

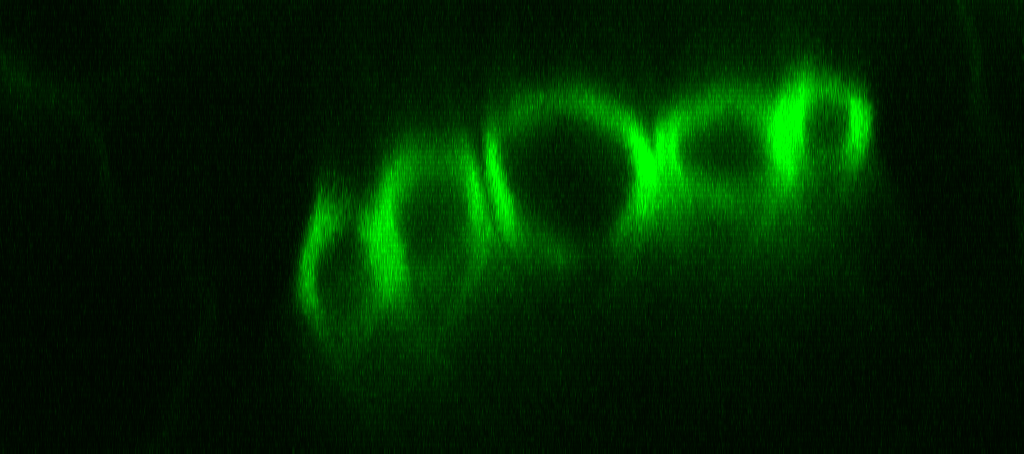

Supplement: Supplementary file 9 — Source data Fig. 1 [file 44318_2024_72_MOESM9_ESM.zip › Figure 1/1A/WT_projection resliced.tif]

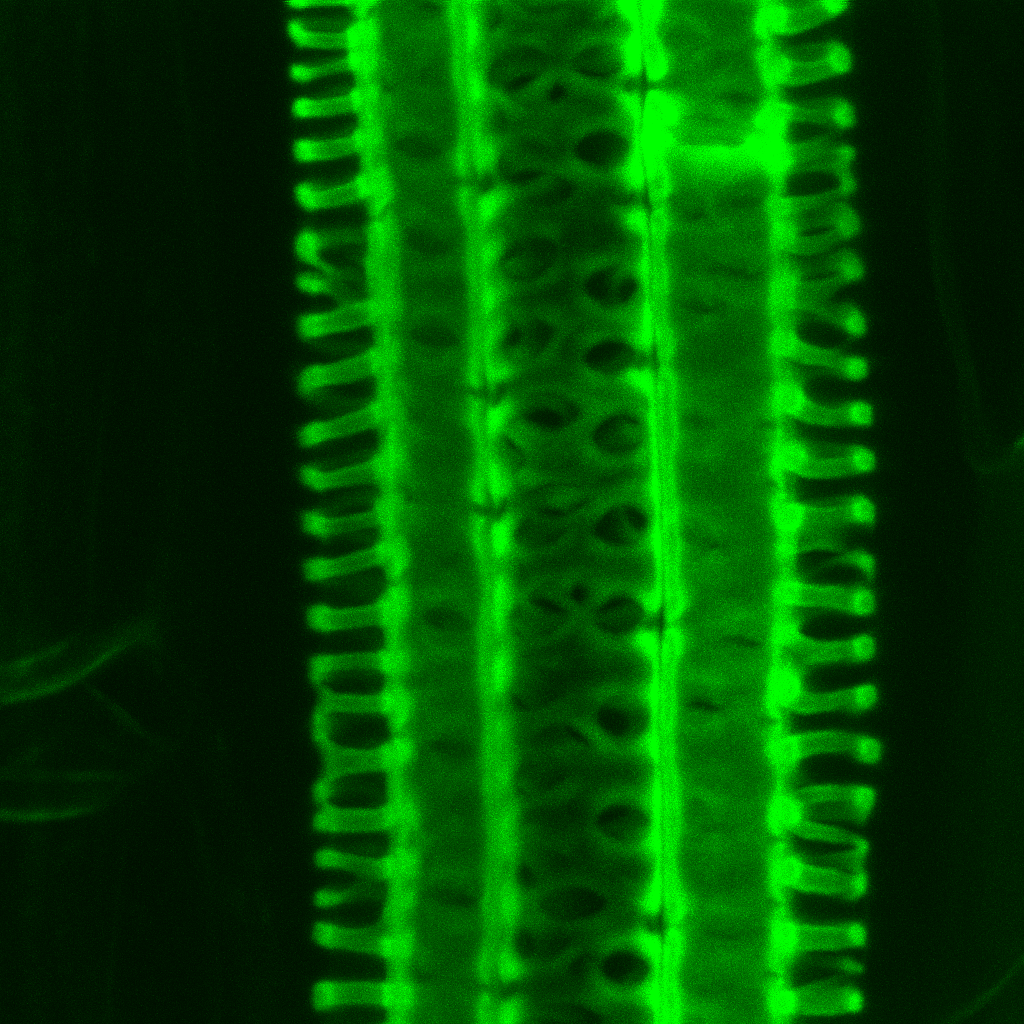

Supplement: Supplementary file 9 — Source data Fig. 1 [file 44318_2024_72_MOESM9_ESM.zip › Figure 1/1A/WT_projection.tif]

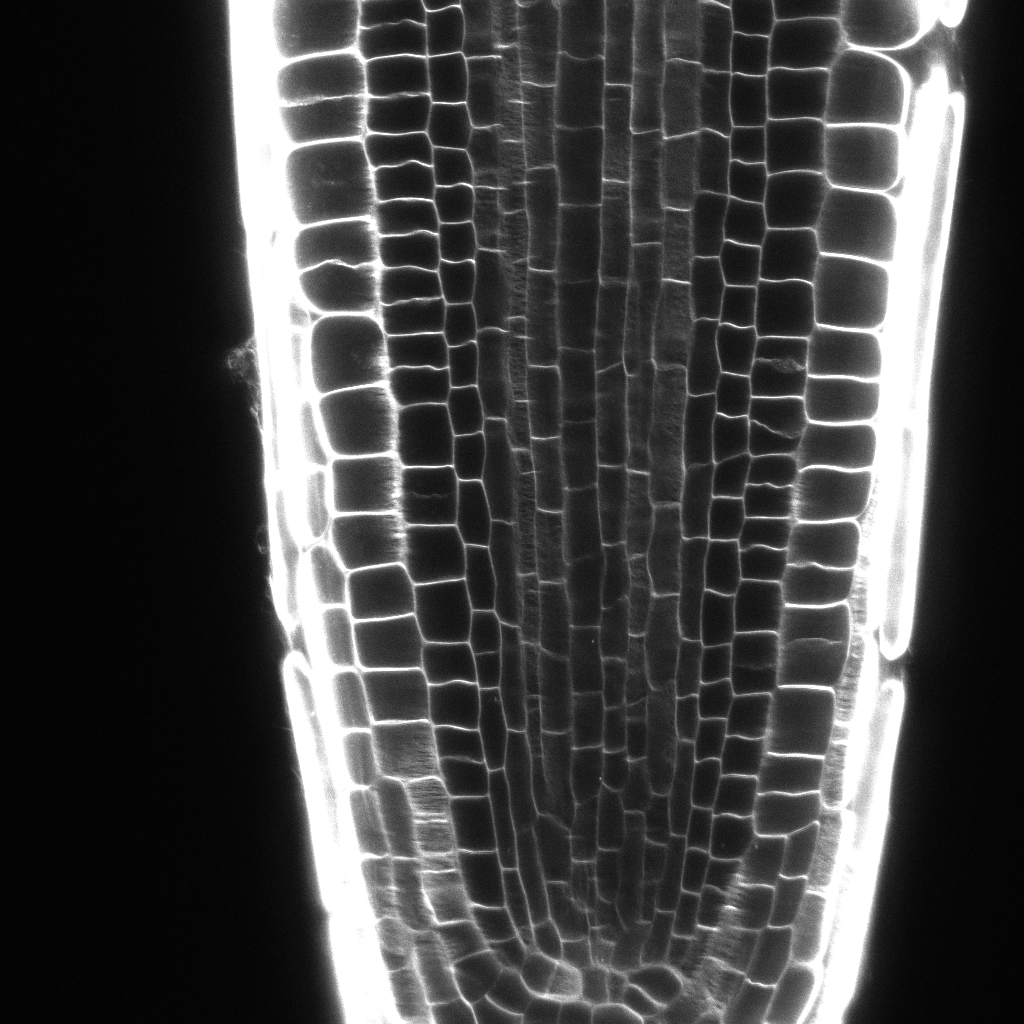

Supplement: Supplementary file 9 — Source data Fig. 1 [file 44318_2024_72_MOESM9_ESM.zip › Figure 1/1E/overview.png]

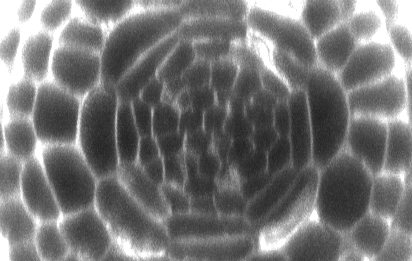

Supplement: Supplementary file 9 — Source data Fig. 1 [file 44318_2024_72_MOESM9_ESM.zip › Figure 1/1E/sgo1_15 μm.tif]

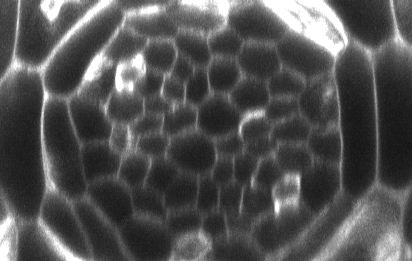

Supplement: Supplementary file 9 — Source data Fig. 1 [file 44318_2024_72_MOESM9_ESM.zip › Figure 1/1E/sgo1_150 μm.tif]

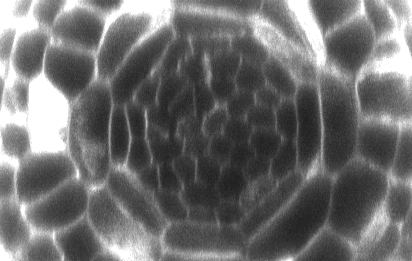

Supplement: Supplementary file 9 — Source data Fig. 1 [file 44318_2024_72_MOESM9_ESM.zip › Figure 1/1E/sgo1_22 μm.tif]

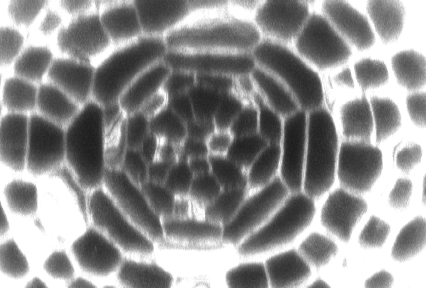

Supplement: Supplementary file 9 — Source data Fig. 1 [file 44318_2024_72_MOESM9_ESM.zip › Figure 1/1E/WT_15 μm.tif]

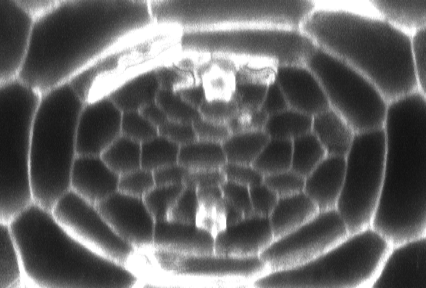

Supplement: Supplementary file 9 — Source data Fig. 1 [file 44318_2024_72_MOESM9_ESM.zip › Figure 1/1E/WT_150 μm.tif]

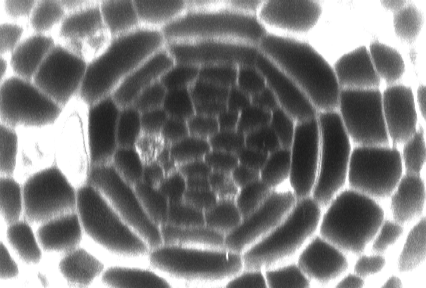

Supplement: Supplementary file 9 — Source data Fig. 1 [file 44318_2024_72_MOESM9_ESM.zip › Figure 1/1E/WT_22 μm.tif]

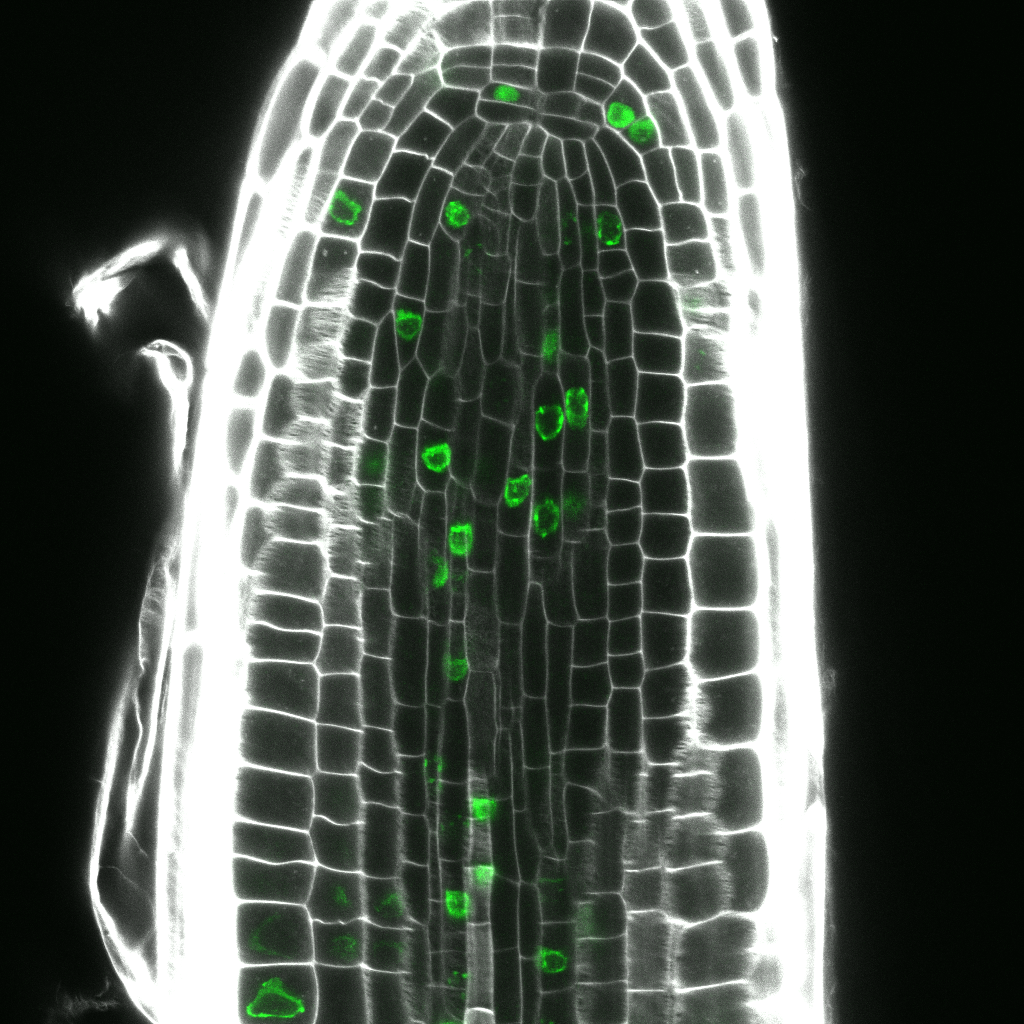

Supplement: Supplementary file 10 — Source data Fig. 2 [file 44318_2024_72_MOESM10_ESM.zip › Figure 2/2A/sgo1_2hpulse.lif - Series001-1.tif (RGB).tif]

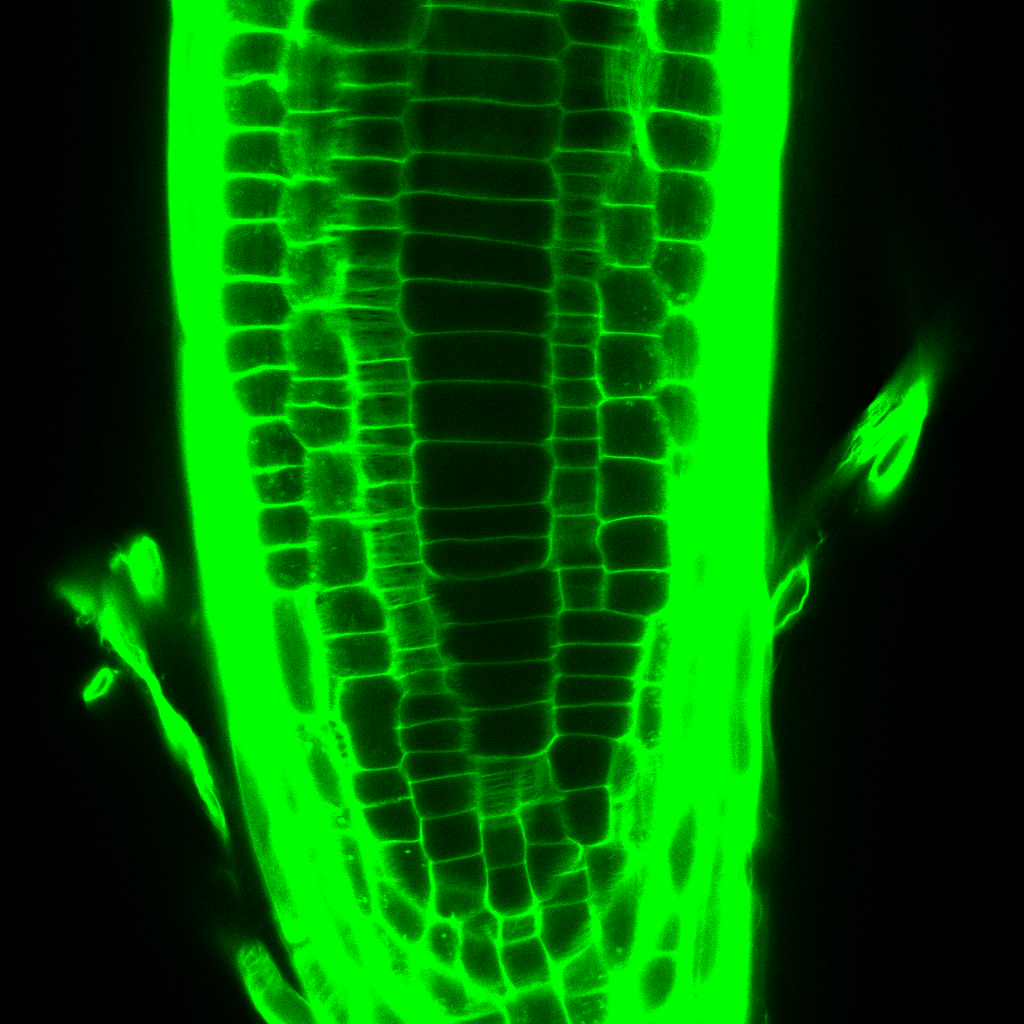

Supplement: Supplementary file 10 — Source data Fig. 2 [file 44318_2024_72_MOESM10_ESM.zip › Figure 2/2C/Col-0_Edu_6hchase.lif - Series009-1.tif]

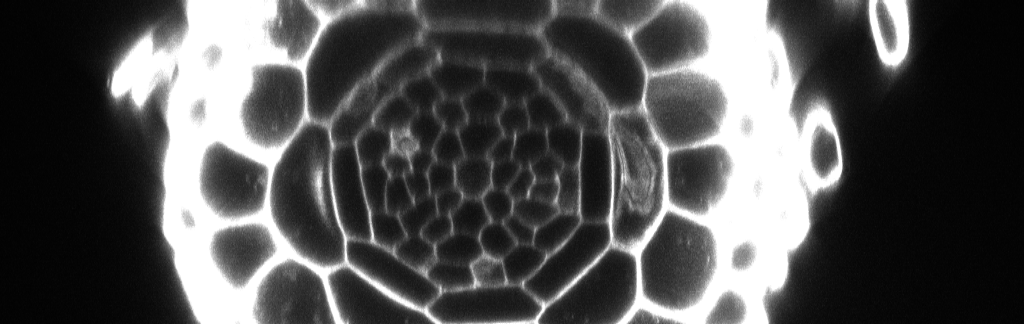

Supplement: Supplementary file 10 — Source data Fig. 2 [file 44318_2024_72_MOESM10_ESM.zip › Figure 2/2D-E/C1-MAX_Reslice of Col-0_Edu_6hchase.lif - Series009-1.tif]

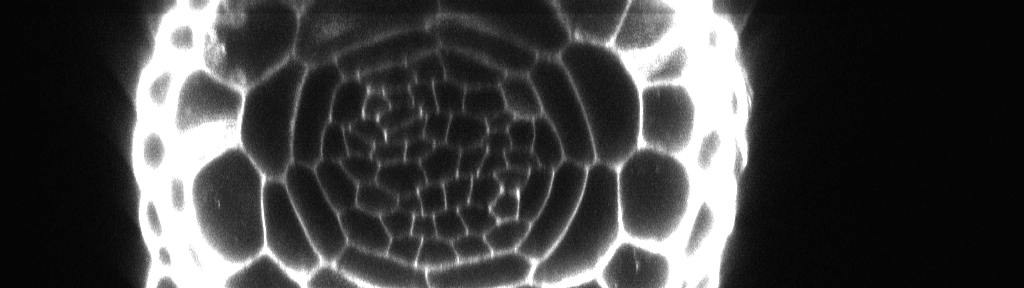

Supplement: Supplementary file 10 — Source data Fig. 2 [file 44318_2024_72_MOESM10_ESM.zip › Figure 2/2D-E/C1-MAX_Reslice of sgo1_EdU_6hchase.lif - Series012-2.tif]

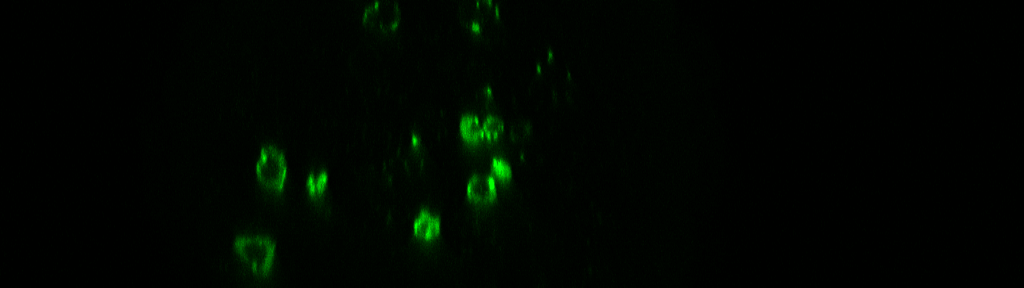

Supplement: Supplementary file 10 — Source data Fig. 2 [file 44318_2024_72_MOESM10_ESM.zip › Figure 2/2D-E/C2-MAX_Reslice of sgo1_EdU_6hchase.lif - Series012-1.png]

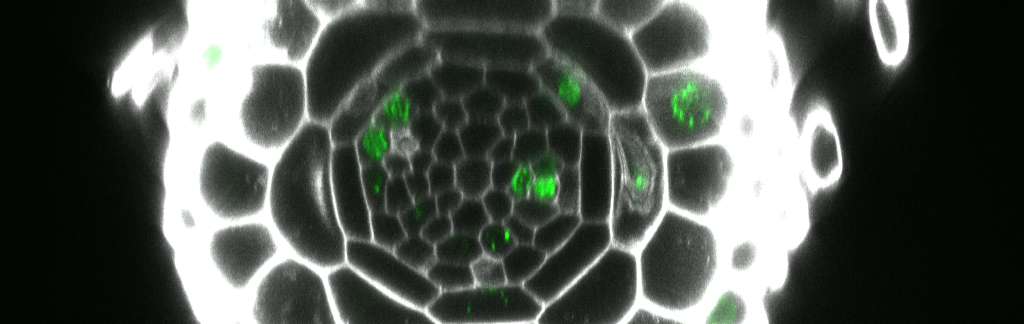

Supplement: Supplementary file 10 — Source data Fig. 2 [file 44318_2024_72_MOESM10_ESM.zip › Figure 2/2D-E/MAX_Reslice of Col-0_Edu_6hchase.lif - Series009-2.tif (RGB).tif]

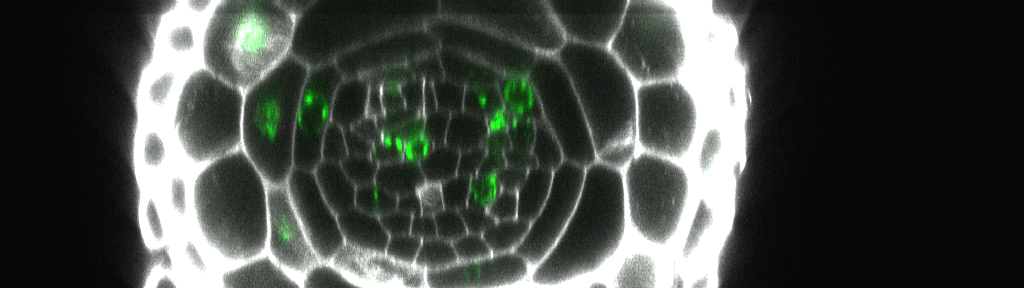

Supplement: Supplementary file 10 — Source data Fig. 2 [file 44318_2024_72_MOESM10_ESM.zip › Figure 2/2D-E/MAX_Reslice of sgo1_EdU_6hchase.lif - Series012-3.tif (RGB).tif]

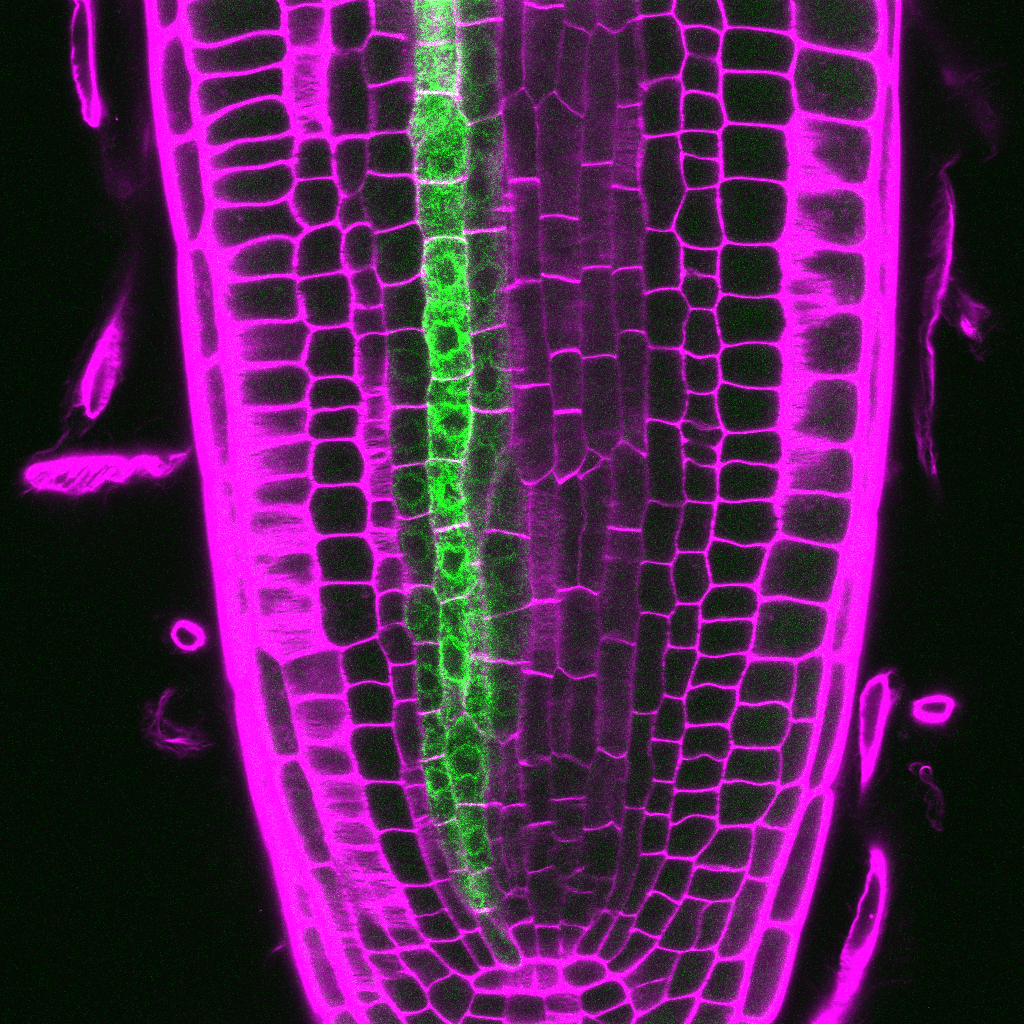

Supplement: Supplementary file 11 — Source data Fig. 3 [file 44318_2024_72_MOESM11_ESM.zip › Figure 3/A/pAHP6erGFP(sgo1).tif]

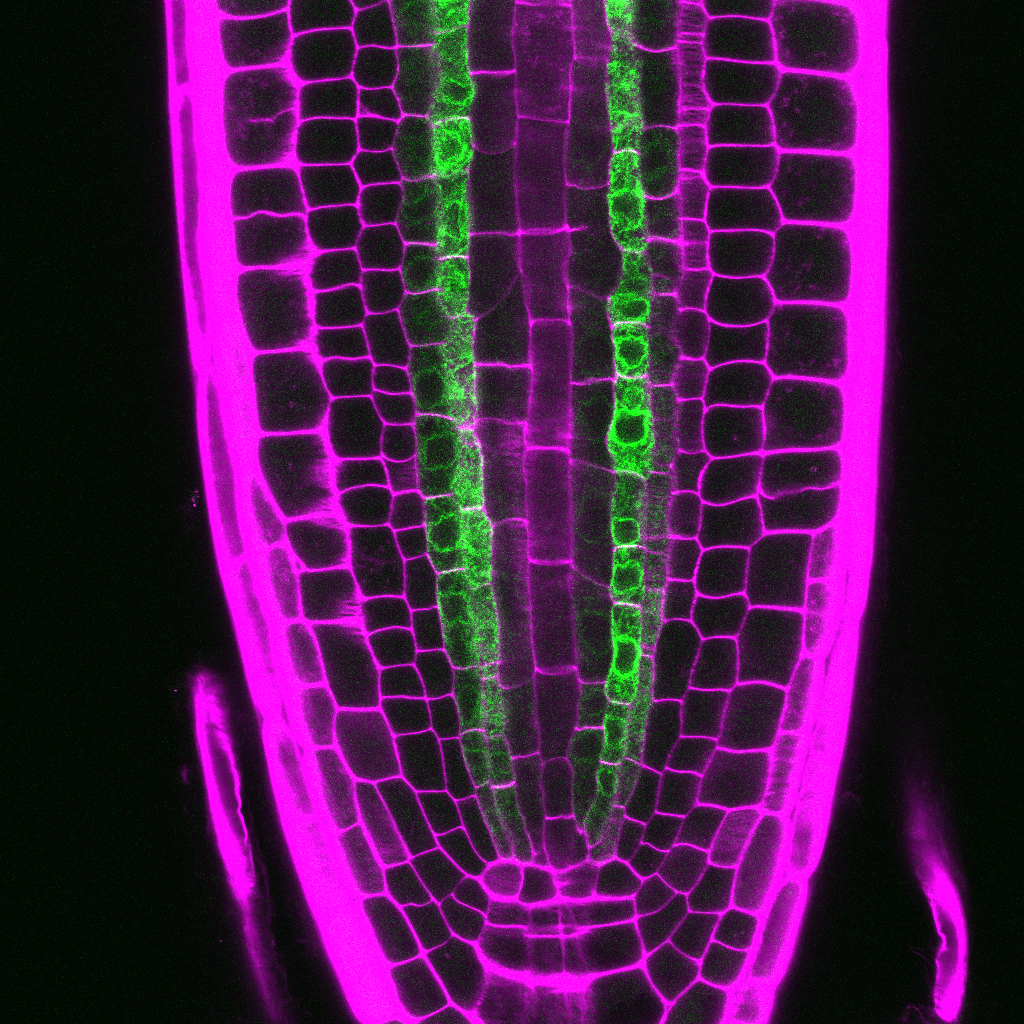

Supplement: Supplementary file 11 — Source data Fig. 3 [file 44318_2024_72_MOESM11_ESM.zip › Figure 3/A/pAHP6erGFP.tif]

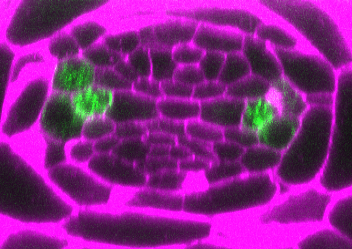

Supplement: Supplementary file 11 — Source data Fig. 3 [file 44318_2024_72_MOESM11_ESM.zip › Figure 3/B/MAX_Reslice pAHP6 WT.tif]

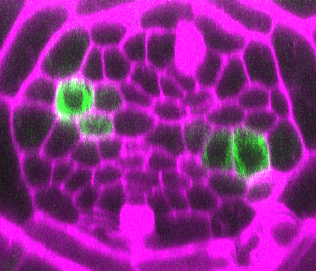

Supplement: Supplementary file 11 — Source data Fig. 3 [file 44318_2024_72_MOESM11_ESM.zip › Figure 3/B/MAX_Reslice pAHP6(sgo1).tif]

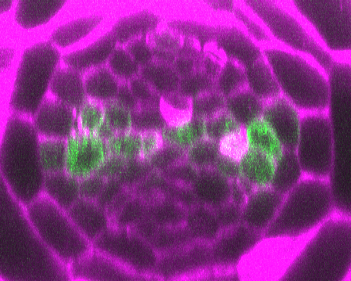

Supplement: Supplementary file 11 — Source data Fig. 3 [file 44318_2024_72_MOESM11_ESM.zip › Figure 3/C/MAX_Reslice of Dr5v2erYFP(sgo1).tif]

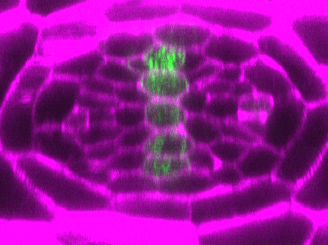

Supplement: Supplementary file 11 — Source data Fig. 3 [file 44318_2024_72_MOESM11_ESM.zip › Figure 3/C/MAX_Reslice of DR5v2erYFP.tif]

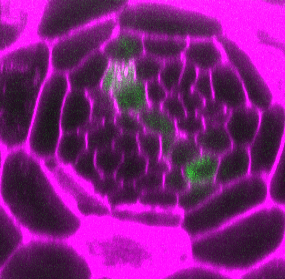

Supplement: Supplementary file 11 — Source data Fig. 3 [file 44318_2024_72_MOESM11_ESM.zip › Figure 3/D/MAX_Reslice of pTMO5-GFP.tif]

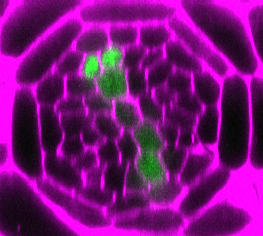

Supplement: Supplementary file 11 — Source data Fig. 3 [file 44318_2024_72_MOESM11_ESM.zip › Figure 3/D/MAX_Reslice ofTMO5(sgo1).tif]

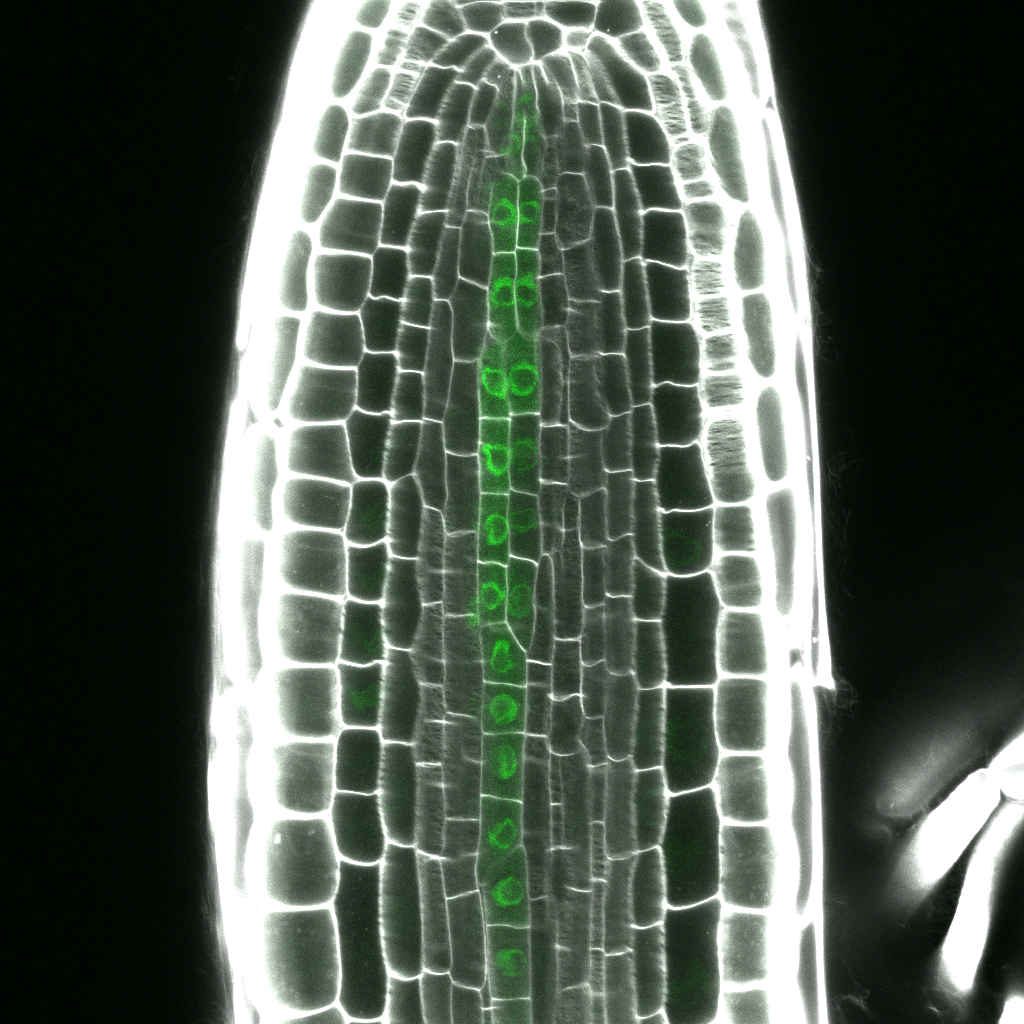

Supplement: Supplementary file 11 — Source data Fig. 3 [file 44318_2024_72_MOESM11_ESM.zip › Figure 3/E/MAX_pTMO5-sgo1.tif]

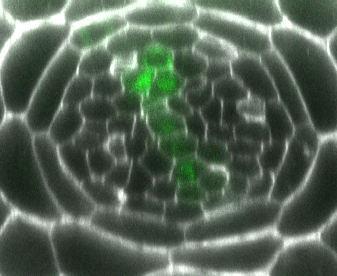

Supplement: Supplementary file 11 — Source data Fig. 3 [file 44318_2024_72_MOESM11_ESM.zip › Figure 3/E/MAX_Reslice of 200519_pTMO5_sgo1.tif]

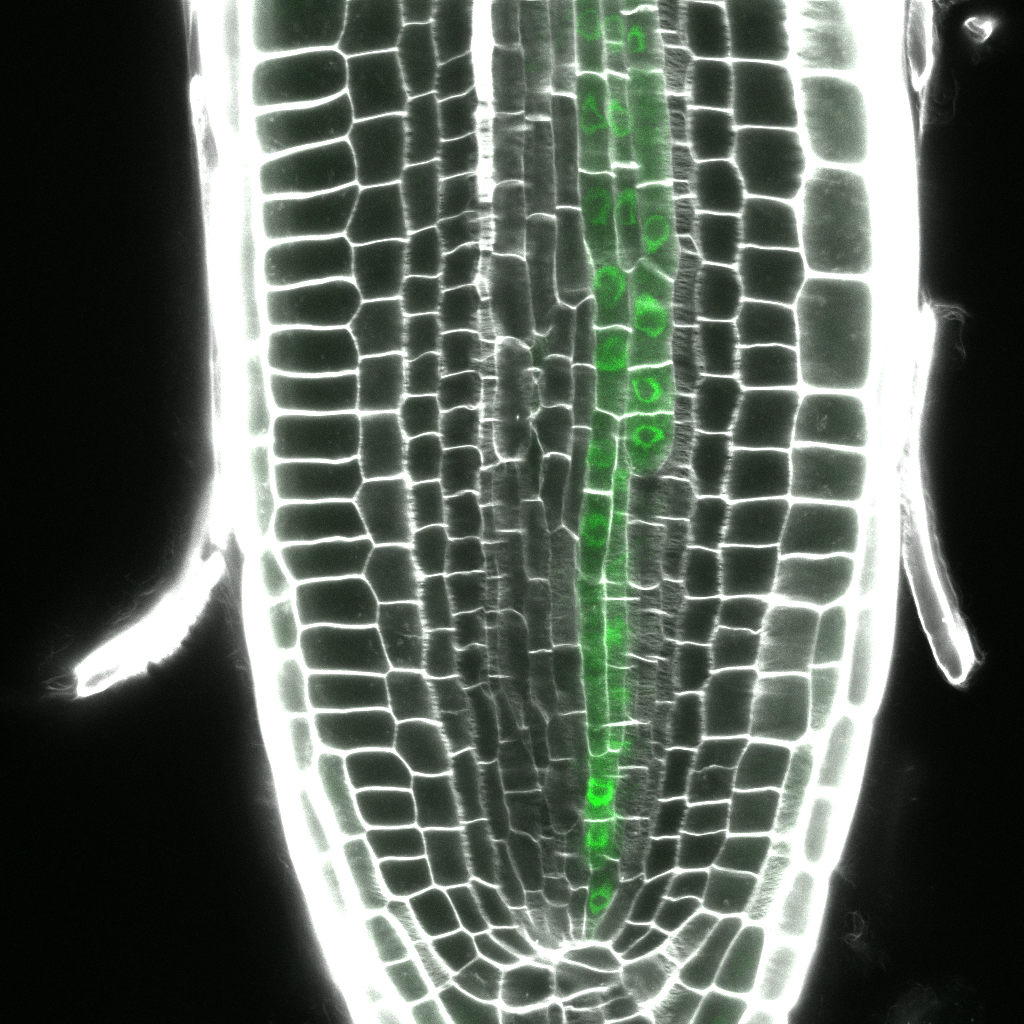

Supplement: Supplementary file 11 — Source data Fig. 3 [file 44318_2024_72_MOESM11_ESM.zip › Figure 3/F/MAX_pTMO5_sgo1.tif]

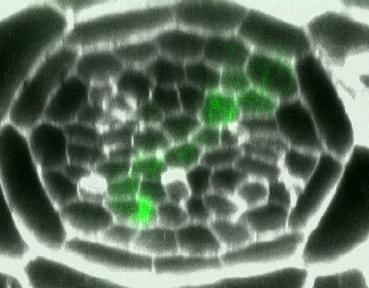

Supplement: Supplementary file 11 — Source data Fig. 3 [file 44318_2024_72_MOESM11_ESM.zip › Figure 3/F/MAX_Reslice of 200430_pTMO5_sgo1.tif]

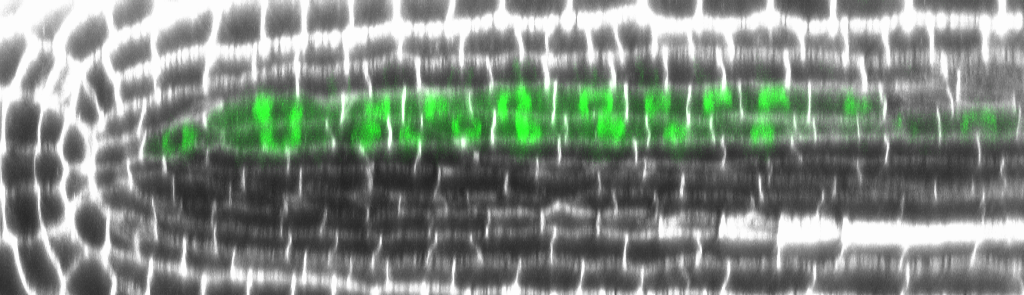

Supplement: Supplementary file 11 — Source data Fig. 3 [file 44318_2024_72_MOESM11_ESM.zip › Figure 3/G/MAX_Reslice of pTMO5_sgo1.tif]

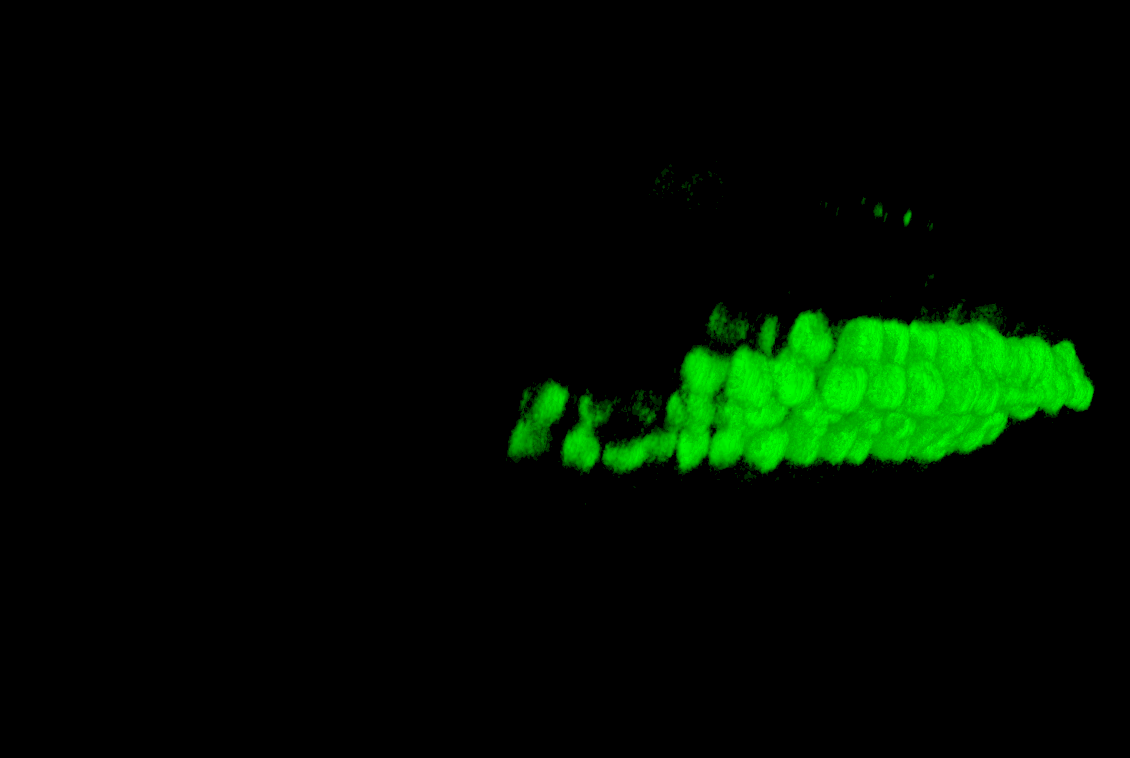

Supplement: Supplementary file 11 — Source data Fig. 3 [file 44318_2024_72_MOESM11_ESM.zip › Figure 3/G/Reslice_200504_Series01_3.png]

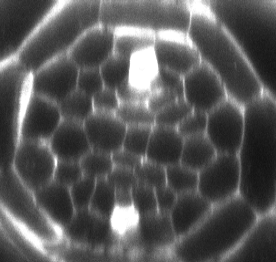

Supplement: Supplementary file 12 — Source data Fig. 4 [file 44318_2024_72_MOESM12_ESM.zip › Figure 4/A/Col-0__150μ.bmp]

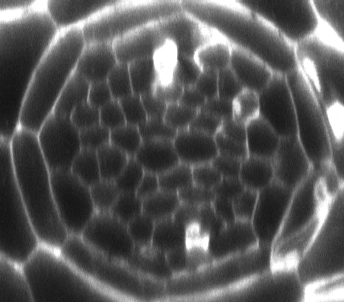

Supplement: Supplementary file 12 — Source data Fig. 4 [file 44318_2024_72_MOESM12_ESM.zip › Figure 4/A/lago10-1_150μ.bmp]

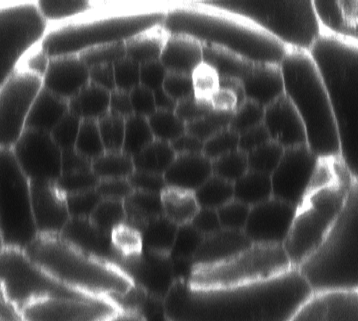

Supplement: Supplementary file 12 — Source data Fig. 4 [file 44318_2024_72_MOESM12_ESM.zip › Figure 4/A/sgo1_150μ.bmp]

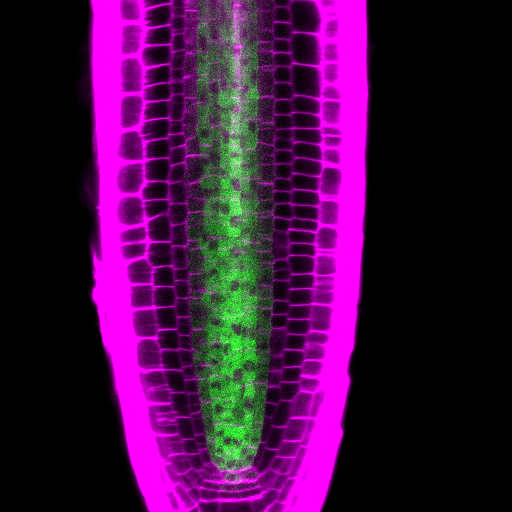

Supplement: Supplementary file 12 — Source data Fig. 4 [file 44318_2024_72_MOESM12_ESM.zip › Figure 4/B/MAX_Ctrl_pAGO10GFP-AGO10_ago10-1_2..tif]

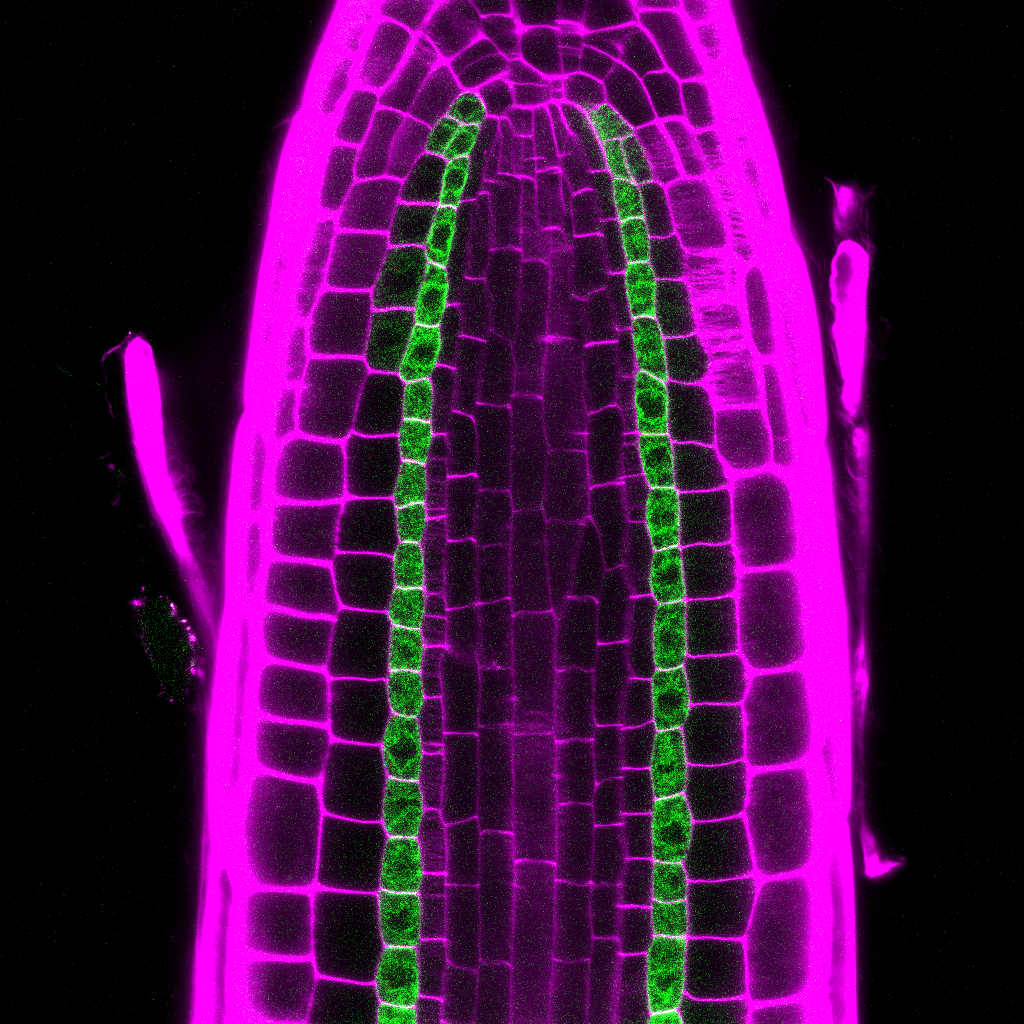

Supplement: Supplementary file 12 — Source data Fig. 4 [file 44318_2024_72_MOESM12_ESM.zip › Figure 4/B/MAX_pMIRNA165-GFP.tif]

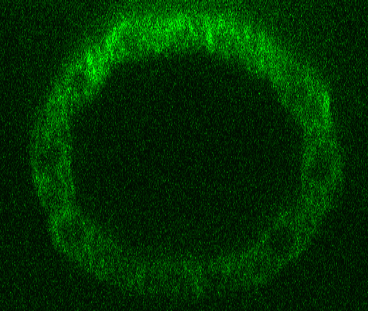

Supplement: Supplementary file 12 — Source data Fig. 4 [file 44318_2024_72_MOESM12_ESM.zip › Figure 4/B/Reslice of 200428_pMIRNA165-GFP.lif - Series002_green_150μ.bmp]

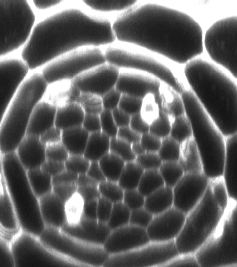

Supplement: Supplementary file 12 — Source data Fig. 4 [file 44318_2024_72_MOESM12_ESM.zip › Figure 4/G/MAX_Reslice of 200314_phbphvcnaer_Series003.tif]

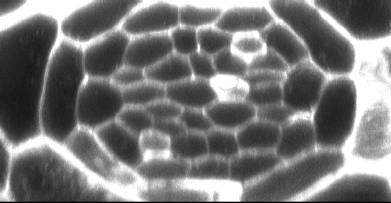

Supplement: Supplementary file 12 — Source data Fig. 4 [file 44318_2024_72_MOESM12_ESM.zip › Figure 4/G/MAX_Reslice of 200505_Ler_Series005.tif]

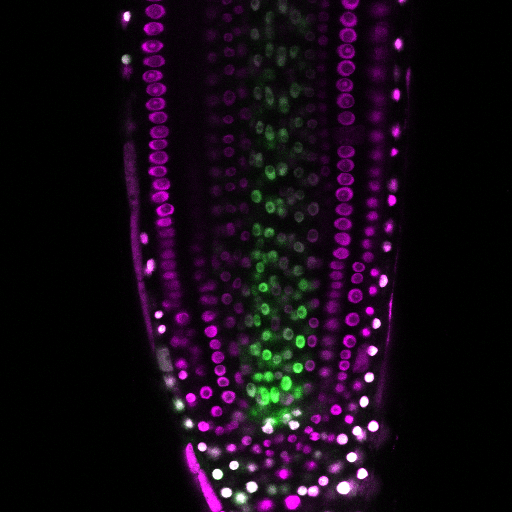

Supplement: Supplementary file 13 — Source data Fig. 5 [file 44318_2024_72_MOESM13_ESM.zip › Figure 5/A/pTCSn(sgo1)_CK_treaktment.tif]

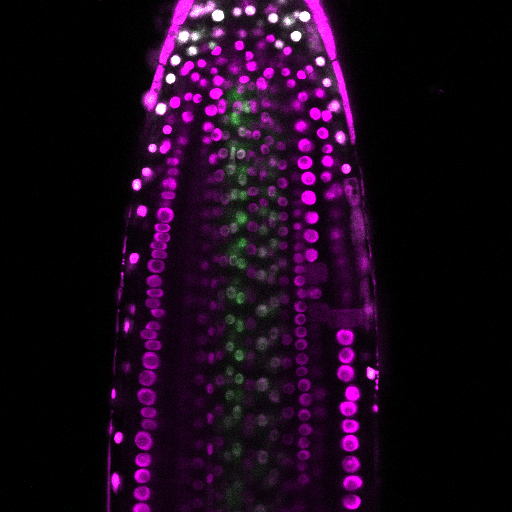

Supplement: Supplementary file 13 — Source data Fig. 5 [file 44318_2024_72_MOESM13_ESM.zip › Figure 5/A/pTCSn(sgo1)_mock.tif]

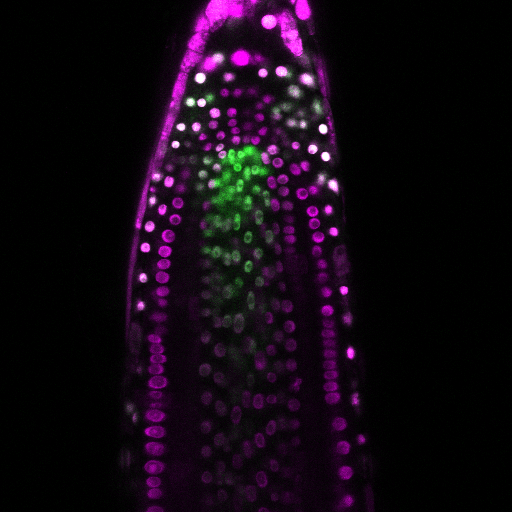

Supplement: Supplementary file 13 — Source data Fig. 5 [file 44318_2024_72_MOESM13_ESM.zip › Figure 5/A/pTCSnGFP_CK_treatment.tif]

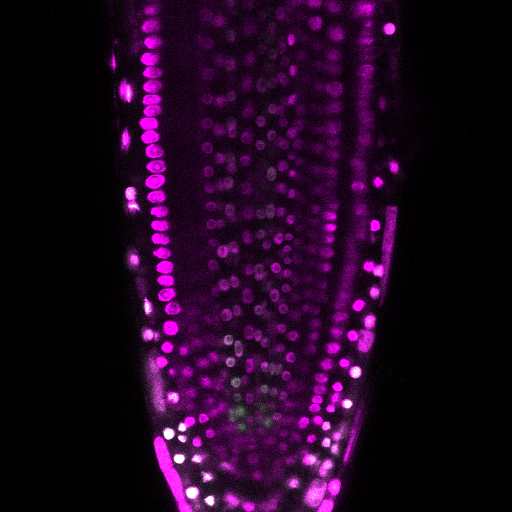

Supplement: Supplementary file 13 — Source data Fig. 5 [file 44318_2024_72_MOESM13_ESM.zip › Figure 5/A/pTCSnGFP_mock.tif]

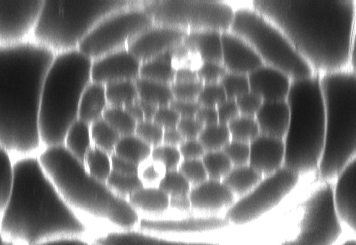

Supplement: Supplementary file 13 — Source data Fig. 5 [file 44318_2024_72_MOESM13_ESM.zip › Figure 5/D/MAX_Reslice of Col-0_1BAP_Series001.tif]

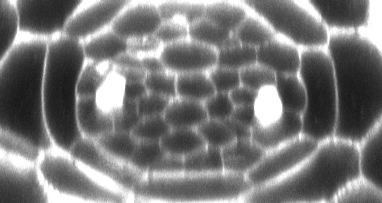

Supplement: Supplementary file 13 — Source data Fig. 5 [file 44318_2024_72_MOESM13_ESM.zip › Figure 5/D/MAX_Reslice of Col-00BAP-Series006.tif]

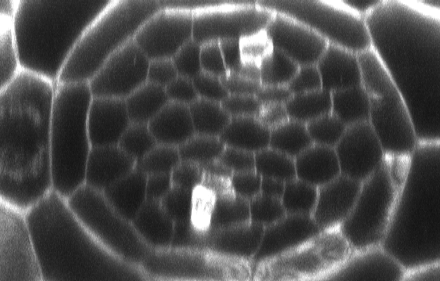

Supplement: Supplementary file 13 — Source data Fig. 5 [file 44318_2024_72_MOESM13_ESM.zip › Figure 5/D/MAX_Reslice of csc1_0BAP_Series006.tif]

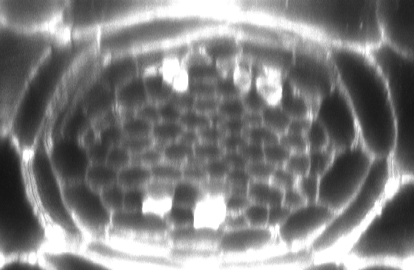

Supplement: Supplementary file 13 — Source data Fig. 5 [file 44318_2024_72_MOESM13_ESM.zip › Figure 5/D/MAX_Reslice of csc1_1BAP_Series004-2.tif]

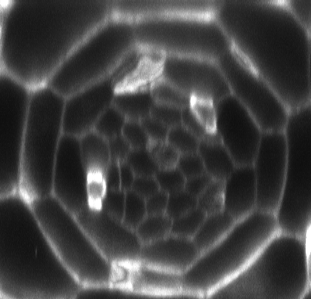

Supplement: Supplementary file 13 — Source data Fig. 5 [file 44318_2024_72_MOESM13_ESM.zip › Figure 5/D/MAX_Reslice of Ler_1BAP_Series002-1.tif]

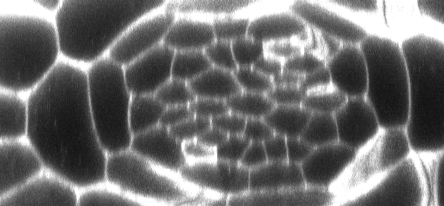

Supplement: Supplementary file 13 — Source data Fig. 5 [file 44318_2024_72_MOESM13_ESM.zip › Figure 5/D/MAX_Reslice of Ler0BAP.lif-Series008.tif]

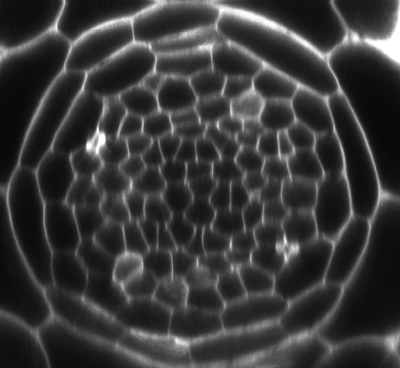

Supplement: Supplementary file 13 — Source data Fig. 5 [file 44318_2024_72_MOESM13_ESM.zip › Figure 5/D/MAX_Reslice of phbphvcna_1BAP-2.tif]

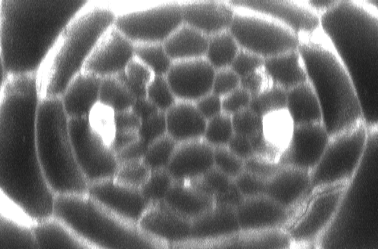

Supplement: Supplementary file 13 — Source data Fig. 5 [file 44318_2024_72_MOESM13_ESM.zip › Figure 5/D/MAX_Reslice of phvphbcnaer 0BAP_Series003.tif]
