# Supplementary material for: ARGONAUTE10 controls cell fate specification and formative cell divisions in the Arabidopsis root
Source: EMBO J. 2024 Apr 2;43(9):7. doi: 10.1038/s44318-024-00072-x (PMC11066080; doi:10.1038/s44318-024-00072-x)
Supplement: Supplementary file 16 — Expanded View Figures [file 44318_2024_72_MOESM16_ESM.pdf]

Expanded View Figures

Figure EV1. Related to Fig. 4. *SGO1* encodes AGO10, which is essential for root vascular patterning in Col-0 and L*er*.

(**A**) Basic fuchsin staining of lignified xylem cells in L*er* and the AGO10 mutant *zll-3*. Upper panels are xy projections of a confocal stack, lower panels optical xy sections through the same stack. Asterisks denote cells with protoxylem differentiation, arrows point to ectopic xylem strands. Scale bar = 25 µm. (**B–D**) Frequency of roots with the indicated number of protoxylem (**B**), metaxylem (**C**) or total xylem (**D**) cells in L*er* and zll-3. Asterisks indicate statistically significant difference from Col-0 based on Mann–Whitney U test (***P* < 0.01, **P* < 0.05). (**C**) Quantification of vascular cell number in cross section of confocal stacks at 15 µm, 22 µm, and 150 µm distance from the quiescent centre (QC) cells in L*er* and *zll-3* meristems. Graph depicts means ± s.d. and individual data ($n = 10–12$). Letters in graph indicate statistically significant differences based on Tukey's post hoc test after one-way ANOVA. (**D**) Frequency of roots with the indicated protoxylem cell number in L*er*, *sgo1* and F1 plants of the indicated crosses, demonstrating that *zll-3* and *sgo1* are allelic to each other. Asterisks indicate statistically significant differences from L*er* based on Dunn's post hoc test with Benjamini–Hochberg correction after Kruskal–Wallis modified U test (***P* < 0.01). (**E**) Immunodetection of AGO10 in Col-0, *sgo1*, and *ago10-1*. Lower panel depicts the Ponceau-stained membrane for loading control.

 © The Author(s)

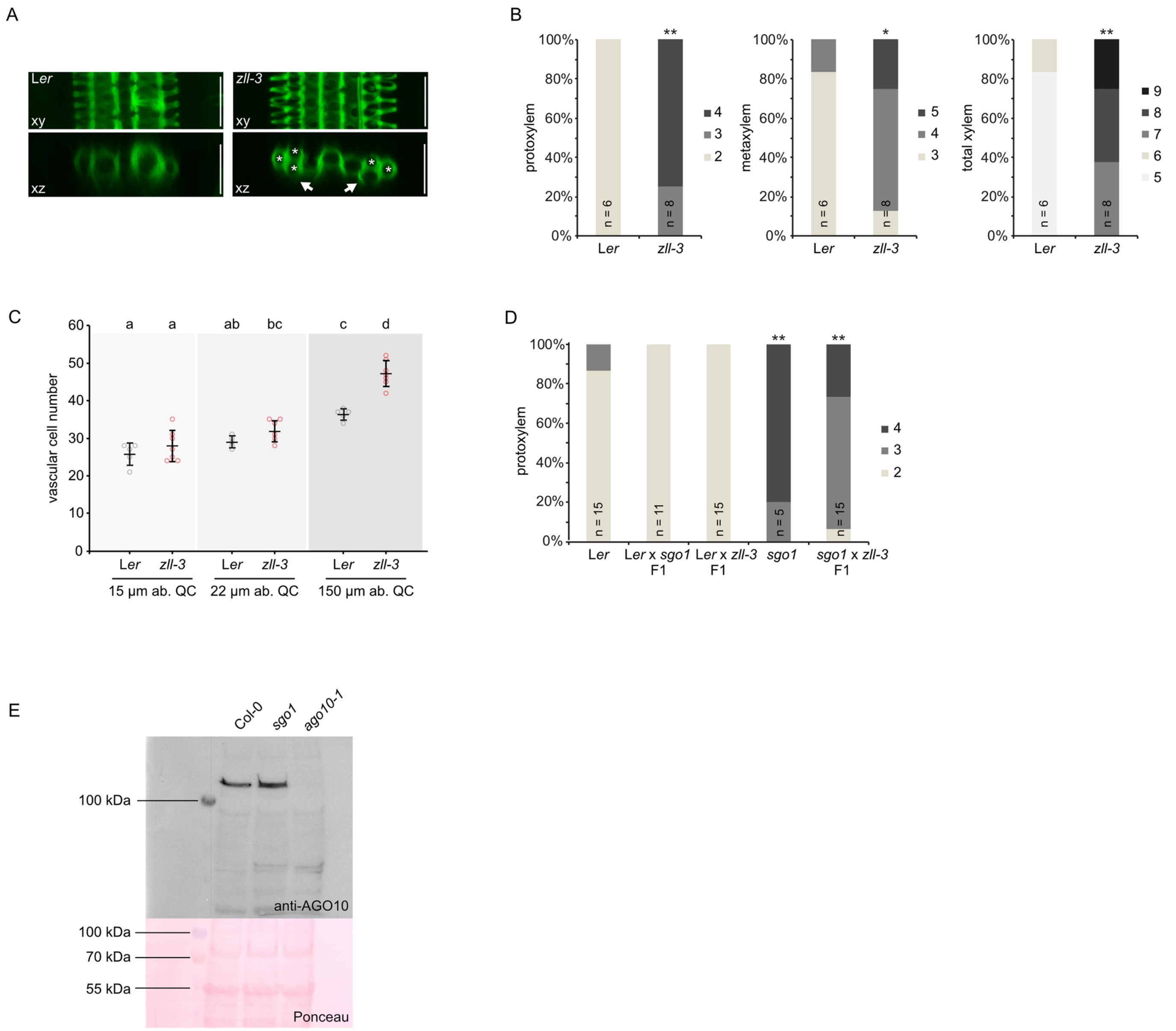
A
Ler
xy
xz
zll-3
xy
xz
B
100%
80%
60%
40%
20%
0%
protoxylem
n = 6
n = 8
**
Ler
zll-3
4
3
2
metaxylem
*
5
4
3
total xylem
9
8
7
6
5
C
60
50
40
30
20
10
0
vascular cell number
a
a
ab
bc
c
d
Ler
zll-3
15 µm ab. QC
22 µm ab. QC
150 µm ab. QC
D
protoxylem
n = 15
n = 11
n = 15
n = 5
n = 15
**
**
Ler
Ler x sgo1 F1
Ler x zll-3 F1
sgo1
sgo1 x zll-3 F1
4
3
2
E
Col-0
sgo1
ago10-1
100 kDa
anti-AGO10
100 kDa
70 kDa
55 kDa
Ponceau

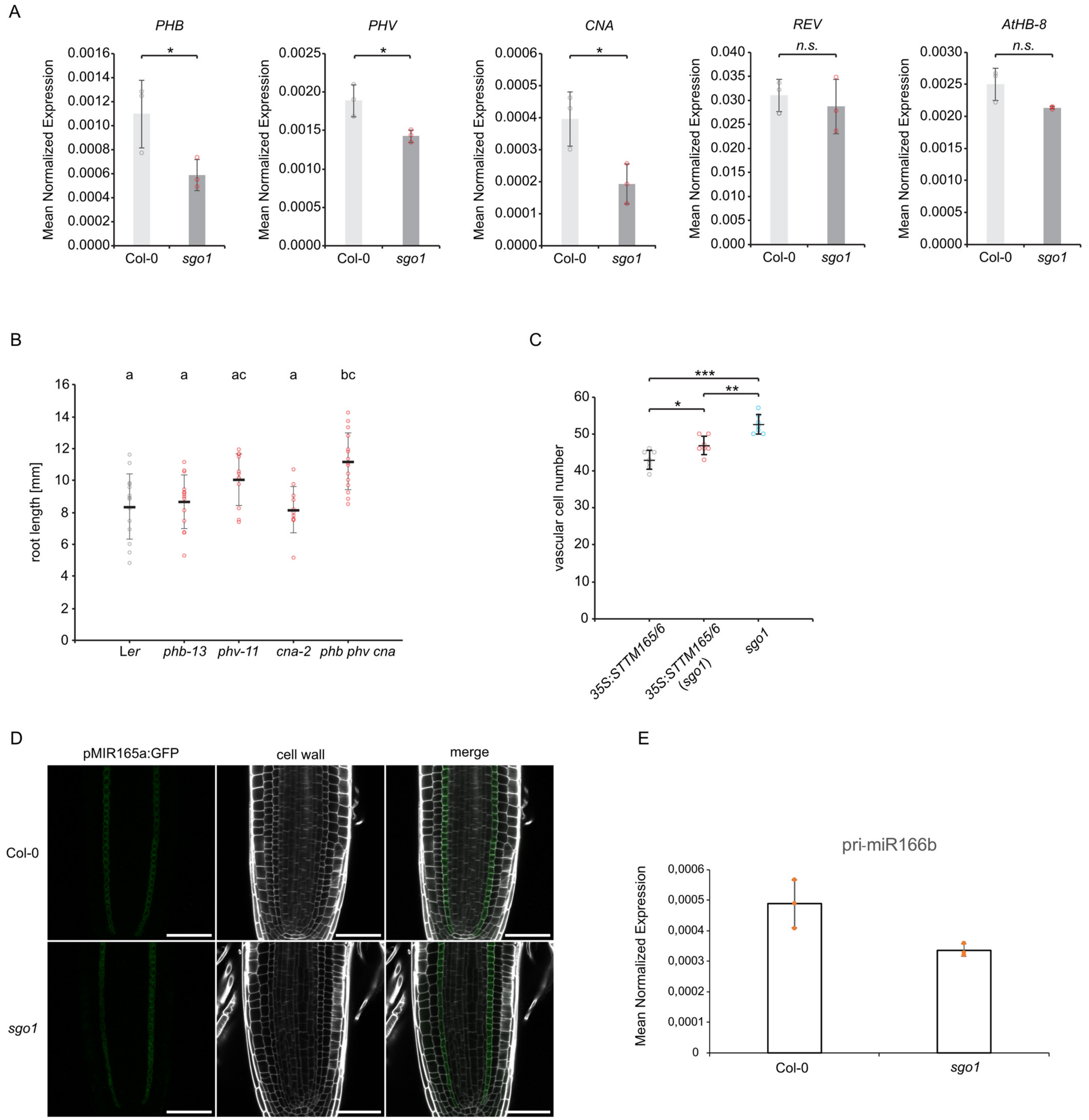

Figure EV2. Related to Fig. 4. AGO10 is required for HD-ZIP III-mediated vascular patterning.

(**A**) Quantitative real-time reverse transcribed PCR (pRT-PCR) analysis indicates reduced HD-ZIP III transcript abundance in *sgo1*. Bars indicate average of Mean normalized expression (MNE) values from three independent biological replicates ± s.d., individual data points are indicated. Asterisks denote statistically significant differences based on a two-tailed student's t-test of log2-transformed MNE values according to Rieu and Powers (Rieu and Powers, 2009). (**B**) Root length quantification of L*er*, *phb-11*, *phv-13*, *cna-2*, and *phb phv cna* 7 days after germination. Graph denotes means ± s.d., individual data points are indicated. Letters indicate statistically significant differences based on Tukey's post hoc test after one-way ANOVA. (**C**) Quantification of vascular cell number in *35S:STTM165/6* expressing a miR165/6 target mimic, *sgo1*, and the target mimic line in the *sgo1* background. Asterisks indicate statistically significant difference based on Tukey's HSD test following one-way ANOVA with (***$P < 0.001$, **$P < 0.01$, *$P < 0.05$). $n = 7$. (**D**) Expression pattern of the pMIR165a:GFP reporter is not altered in the *sgo1* background. Scale bars = 50 μm. (**E**) Levels of the pri-miR165/6 transcripts are not significantly altered in the *sgo1* background. QPCR results with a primer pair binding all pri-miR transcripts are shown. Bars indicate average of Mean normalized expression values from three independent biological replicates ± s.d., individual data points are indicated.

 © The Author(s)

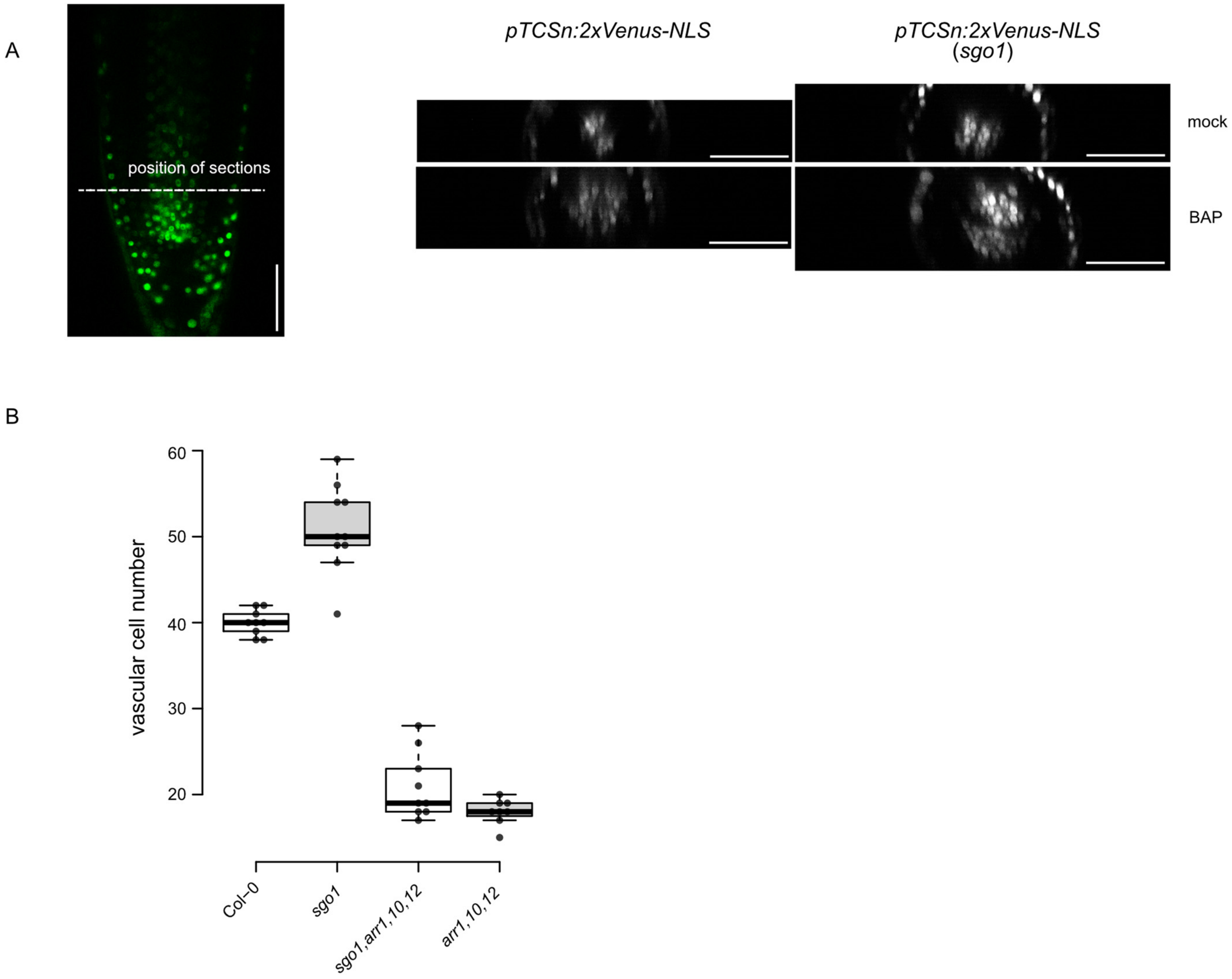

Figure EV3. Related to Fig. 5. Increased vascular cell numbers in sgo1 depend on intact cytokinin signalling.

(**A**) Confocal sections of *pTCSn:2xVenus-NLS pUB10:3xmCherry-NLS* marker line after mock treatment or after growth on medium supplemented with 0.1 µM BAP. Scale bar = 50 µm. (**B**) Lesion of three of the main type-B ARR CK response regulators, ARR1, ARR10, and ARR12 (Argyros et al, 2008), results in a dramatic reduction of vascular cell numbers in both the Col-0 and *sgo1* background, respectively. Box plots depict median and upper and lower quartile (boxes), average (cross) and data range (whiskers), excluding outliers, of root length under the indicated conditions, dots represent individual data points. $n = 8$–10.

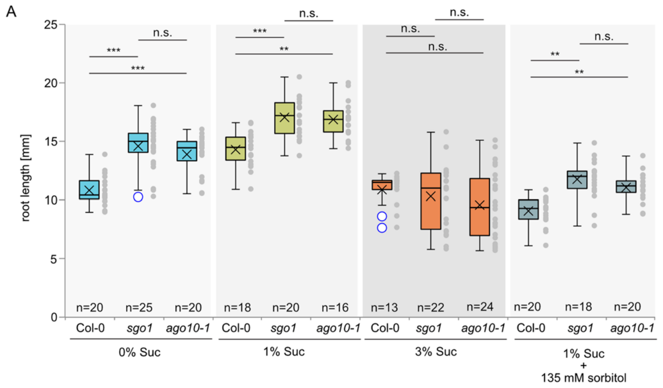

Figure EV4. Related to Fig. 6. AGO10 is required for phenotypic robustness.

(**A**) Root length of Col-0, *sgo1*, and *ago10-1* at 6DAG under the indicated conditions. Box plots depict median and upper and lower quartile (boxes), average (cross) and data range (whiskers) of root length under the indicated conditions, dots represent individual data points. Asterisks indicate statistically significant difference based on Tukey's HSD test following one-way ANOVA with (***$P < 0.001$, **$P < 0.01$, n.s. = not significant). Significance differences are only indicated within a given condition.

 © The Author(s)

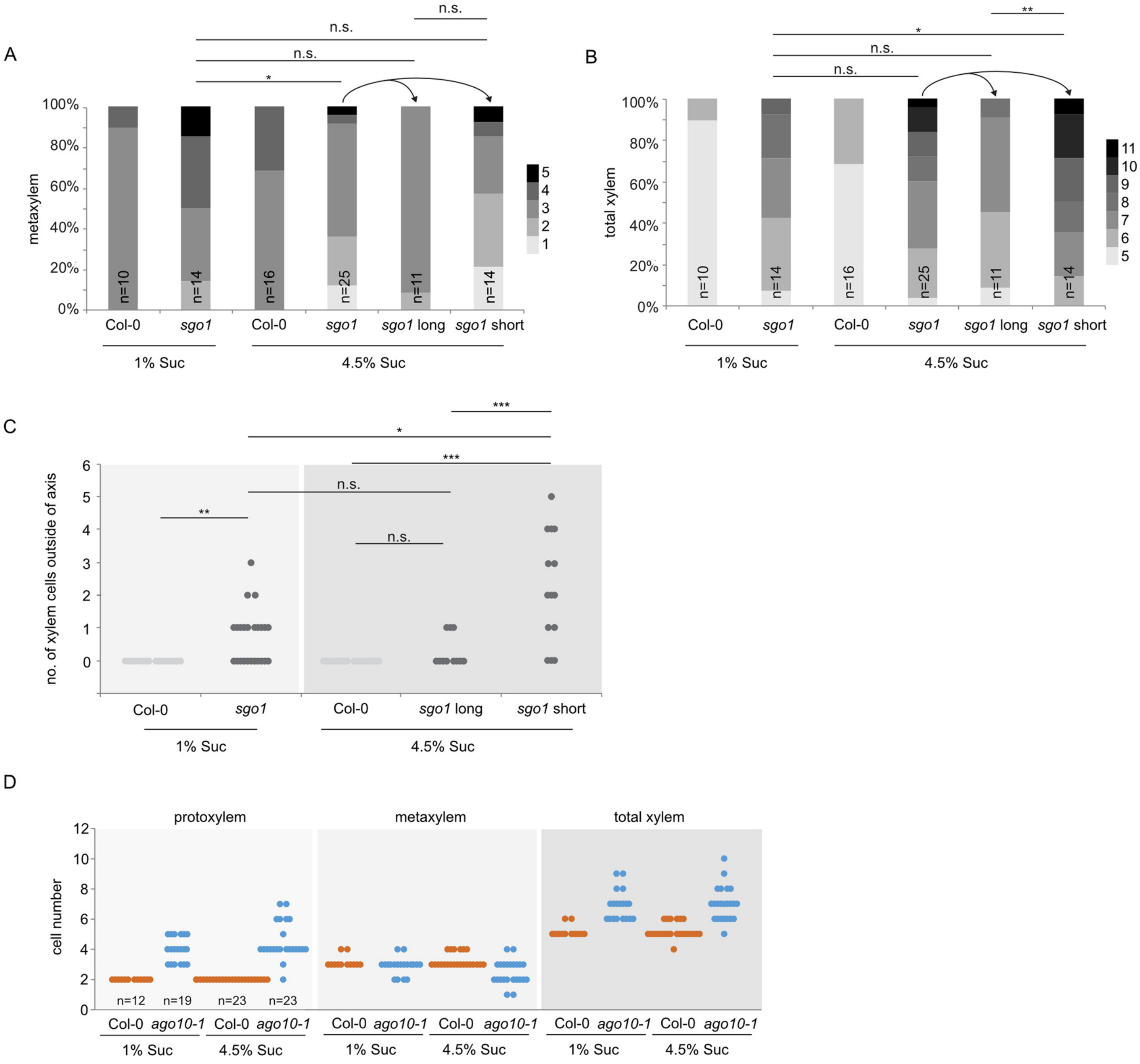

Figure EV5. Related to Fig. 6. AGO10 is required for phenotypic robustness.

(**A**, **B**) Frequency of roots with the indicated number of metaxylem (**A**) and total xylem (**B**) of Col-0 and *sgo1* grown on 1% or 4.5% sucrose. Short and long *sgo1* roots on 4.5% sucrose are depicted separately and combined. Asterisks indicate statistically significant difference based on Mann–Whitney U test (***P* < 0.01, **P* < 0.05, n.s. = not significant). (**C**) Plot of individual roots grown under the indicated conditions according to the number of ectopic xylem cells differentiating outside of the xylem axis, i.e. in procambial position. Asterisks indicate statistically significant differences based on Dunn's post hoc test with Benjamini–Hochberg correction after Kruskal–Wallis modified U test (****P* < 0.001, ***P* < 0.01, **P* < 0.05, n.s. = not significant). (**D**) Plot of differentiated xylem cell number of Col-0 and *ago10-1* roots grown under the indicated conditions.

© The Author(s)
